# Light, Ethylene and Auxin Signaling Interaction Regulates Carotenoid Biosynthesis During Tomato Fruit Ripening

**DOI:** 10.3389/fpls.2018.01370

**Published:** 2018-09-18

**Authors:** Aline Bertinatto Cruz, Ricardo Ernesto Bianchetti, Frederico Rocha Rodrigues Alves, Eduardo Purgatto, Lazaro Eustaquio Pereira Peres, Magdalena Rossi, Luciano Freschi

**Affiliations:** ^1^Departamento de Botânica, Instituto de Biociências, Universidade de São Paulo, São Paulo, Brazil; ^2^Departamento de Alimentos e Nutrição Experimental, Faculdade de Ciências Farmacêuticas, Universidade de São Paulo, São Paulo, Brazil; ^3^Departamento de Ciências Biológicas, Escola Superior de Agricultura “Luiz de Queiroz", Universidade de São Paulo, Piracicaba, Brazil

**Keywords:** fruit ripening, auxin, ethylene, photomorhogenesis, tomato, climacteric, high pigment mutant, light–dark

## Abstract

Light signaling and plant hormones, particularly ethylene and auxins, have been identified as important regulators of carotenoid biosynthesis during tomato fruit ripening. However, whether and how the light and hormonal signaling cascades crosstalk to control this metabolic route remain poorly elucidated. Here, the potential involvement of ethylene and auxins in the light-mediated regulation of tomato fruit carotenogenesis was investigated by comparing the impacts of light treatments and the light-hyperresponsive *high pigment-2* (*hp2*) mutation on both carotenoid synthesis and hormonal signaling. Under either light or dark conditions, the overaccumulation of carotenoids in *hp2* ripening fruits was associated with disturbed ethylene production, increased expression of genes encoding master regulators of ripening and higher ethylene sensitivity and signaling output. The increased ethylene sensitivity observed in *hp2* fruits was associated with the differential expression of genes encoding ethylene receptors and downstream signaling transduction elements, including the downregulation of the transcription factor *ETHYLENE RESPONSE FACTOR.E4*, a repressor of carotenoid synthesis. Accordingly, treatments with exogenous ethylene promoted carotenoid biosynthetic genes more intensively in *hp2* than in wild-type fruits. Moreover, the loss of *HP2* function drastically altered auxin signaling in tomato fruits, resulting in higher activation of the auxin-responsive promoter *DR5*, severe down-regulation of *AUXIN/INDOLE-3-ACETIC ACID* (*Aux/IAA*) genes and altered accumulation of *AUXIN RESPONSE FACTOR* (*ARF*) transcripts. Both tomato *ARF2* paralogues (*Sl-ARF2a* and *SlARF2b*) were up-regulated in *hp2* fruits, which agrees with the promotive roles played by these ARFs in tomato fruit ripening and carotenoid biosynthesis. Among the genes differentially expressed in *hp2* fruits, the additive effect of light treatment and loss of *HP2* function was particularly evident for those encoding carotenoid biosynthetic enzymes, ethylene-related transcription factors, Aux/IAAs and ARFs. Altogether, the data uncover the involvement of ethylene and auxin as part of the light signaling cascades controlling tomato fruit metabolism and provide a new link between light signaling, plant hormone sensitivity and carotenoid metabolism in ripening fruits.

## Introduction

Light plays a dual role during plant development, providing energy for photosynthesis and information for adjusting plant growth, development and reproduction. Processes as diverse as seed germination, seedling de-etiolation, phototropism, flowering, fruit pigmentation and entrainment of circadian rhythms are intrinsically regulated by light stimuli (Azari et al., 2010a; Llorente et al., 2016a). In tomato, a model crop for fleshy fruits, multiple photomorphogenic mutants have been identified over the years, greatly facilitating the deciphering of the relevance of light signaling in fruit biology and quality traits (Levin et al., 2006; Azari et al., 2010b). Among these genotypes, the tomato *high pigment* (*hp*) mutants *hp1* and *hp2* have been instrumental in illustrating the positive role of light signaling in fruit metabolism and nutritional composition. These mutants are characterized by their exaggerated light responsiveness, over-accumulation of chlorophyll and chloroplasts in leaves and immature fruits as well as intense red fruit pigmentation (Mustilli et al., 1999; Levin et al., 2003, 2006). Compared to their WT counterparts, *hp* ripe fruits display higher levels of health-promoting substances, including carotenoids, flavonoids, tocopherol (vitamin E) and ascorbic acid (vitamin C) (Yen et al., 1997; Liu et al., 2004; Kolotilin et al., 2007). Fruit carotenogenesis is particularly up-regulated in *hp* mutants, which agrees with the positive influence of light on isoprenoid metabolism in both fruit and vegetative tissues (Piringer and Heinze, 1954; Alba et al., 2000; Schofield and Paliyath, 2005).

Genetic analysis of *hp1* and *hp2* alleles revealed mutations in tomato homologs of the nuclear proteins UV-DAMAGED DNA BINDING PROTEIN1 (DDB1) and DEETIOLATED1 (DET1), respectively, two negative regulators of light signal transduction (Mustilli et al., 1999; Schroeder et al., 2002; Levin et al., 2003; Lieberman et al., 2004; Liu et al., 2004). Confirming these findings, silencing of *Sl-DDB1/HP1* or *Sl-DET1/HP2* greatly promotes plastid biogenesis and carotenoid accumulation in fruit tissues (Davuluri et al., 2004, 2005; Wang et al., 2008). Besides *Sl-DDB1/HP1* and *Sl-DET1/HP2*, other components of the light signaling cascade have also been implicated in controlling tomato fruit metabolism, including the E3 ubiquitin-ligases CULLIN4 (CUL4) and CONSTITUTIVELY PHOTOMORPHOGENIC 1 (COP1), as well as the transcription factors ELONGATED HYPOCOTYL 5 (HY5) and PHYTOCHROME-INTERACTING FACTORs (PIFs) (Liu et al., 2004; Davuluri et al., 2005; Wang et al., 2008; Llorente et al., 2016b). Constitutive silencing of tomato *CUL4*, *COP1* or *PIF1a* generates fruits with increased carotenoid levels (Liu et al., 2004; Wang et al., 2008; Llorente et al., 2016b), whereas the opposite phenotype is caused by the suppression of the light-signaling effector *HY5* (Liu et al., 2014). Significant alterations in carotenoid biosynthesis have also been observed in ripening fruits of transgenic plants with fruit-specific silencing of phytochrome (PHY)-encoding genes (Bianchetti et al., 2018), as well as in cryptochrome1a (CRY1a)-deficient mutants and *CRY1a*-overexpressing lines (Liu et al., 2018).

Virtually all fruit metabolic processes influenced by light are also strictly controlled by an integrated, multi-hormonal signaling network (Giovannoni, 2004; Karlova et al., 2014; Liu M. et al., 2015). Compelling data implicate ethylene as a primary regulator of multiple ripening-related physiological, biochemical, and molecular processes (Barry and Giovannoni, 2007; Pech et al., 2012). Accordingly, disturbed ethylene biosynthesis, perception or signal transduction directly impact fruit ripening initiation and progression (Liu M. et al., 2015). Without undermining the role of ethylene, auxins have also been shown to interfere with fruit ripening and carotenoid accumulation, as revealed by the delayed ripening phenotype and the down-regulation in carotenoid biosynthesis observed in IAA-treated tomato fruits (Su et al., 2015).

Although light signaling and plant hormones, such as ethylene and auxins, are essential regulators of tomato fruit ripening and carotenogenesis, whether and how the light and hormonal signaling cascades crosstalk to control these metabolic processes remains poorly elucidated. Here, the potential involvement of ethylene and auxins in the light-mediated regulation of tomato fruit ripening and carotenogenesis was investigated by comparing the impact of light and dark treatments, isolated or combined with the loss of *Sl-DET1*/*HP2* function, on both carotenoid synthesis and hormonal signaling.

## Materials and Methods

### Plant Material and Growth Conditions

Wild-type (WT) *Solanum lycopersicum* L. (cv. Micro-Tom), a near-isogenic line (NIL) harboring the mutation *high pigment-2* (*hp2*), and transgenic plants carrying the synthetic auxin-responsive (*DR5*) or ethylene-responsive (*EBS*) promoters fused to the reporter gene *uid* (encoding a β-glucuronidase, GUS) were obtained from the tomato mutant collection maintained at ESALQ, University of São Paulo (USP), Brazil (Carvalho et al., 2011). Crosses and successive screening were performed to generate the double mutants *hp2-DR5::GUS* and *hp2-EBS::GUS.* Plants were grown in 6-L rectangular pots containing a 1:1 mixture of commercial substrate (Plantmax HT, Eucatex, São Paulo, Brazil) and expanded vermiculite, supplemented with 1 g L^-1^ of NPK 10:10:10, 4 g L^-1^ of dolomite limestone (MgCO_3_ + CaCO_3_) and 2 g L^-1^ thermophosphate (Yoorin Master^®^, Yoorin Fertilizantes, Brazil) in greenhouse under automatic irrigation at an average mean temperature of 25°C, 11.5 h/13 h (winter/summer) photoperiod and approximately 250–350 μmol m^-2^ s^-1^ PAR irradiance.

### Light Treatments

Fruits at mature green (MG) stage were harvested about 30 days after anthesis (dpa) and transferred to continuous white light or maintained under absolute darkness (D) until reaching distinct ripening stages. White light was delivered at around 50 μmol m^-2^ s^-1^ and supplied by an array of SMD5050 Samsung LEDs mounted in a temperature-controlled growth chamber maintained at 25 ± 1°C and air relative humidity at 80 ± 5%. Top and bottom illumination were applied to homogenize the light environment surrounding the fruits. Fruits were placed into a 0.5-L sealed transparent vessel and continuously flushed with ethylene-free, humidified air (1 L min^-1^) to avoid accumulation of ET inside the containers. Samples from light- or dark-incubated fruits were harvested under white light or dim green light, respectively. Harvesting was performed at the same daytime to avoid possible fluctuations in the parameters due to circadian rhythm. Pericarp samples (without seeds, columella, placental tissues and locule walls) were harvested as soon as the fruits had reached the following ripening stages: MG (displaying jelly placental tissues, 2 days after harvesting), Bk (breaker, showing the first external yellow color signals) and Bk1, Bk3, Bk6, and Bk12, corresponding 1, 3, 6, and 12 days after Bk, respectively. Fruits at distinct treatments achieved each ripening stage at a different number of days of treatment. Four biological samples composed of at least five fruits each were harvested at each sampling time. Ethylene emission analysis and quantitative *in vitro* GUS activity assays were performed immediately after harvesting. For all other analyses, samples were frozen in liquid N_2_, powdered and stored at -80°C until use.

### Hormonal Treatments

Fruits harvested at the MG stage were submitted to ethylene or auxin treatment at 25°C in the presence of white light (50 μmol m^-2^ s^-1^). For the ethylene treatment, fruits were kept inside transparent sealed tubes in the presence of 50 ppm of ethylene, whereas control fruits were maintained in ethylene-free air. For the auxin treatment, fruits were injected with a buffer solution containing 10 mM 2-(*N*-morpholino) ethanesulfonic acid (MES) pH 5.6, 3% sorbitol (w/v) and 100 μM of indole-3-acetic acid (IAA) whereas control fruits were treated with buffer without IAA (Su et al., 2015). After 6 h treatment, fruit pericarp samples were collected before snap freezing in nitrogen.

### Chlorophyll Quantification and Carotenoid Profile

Chlorophyll extraction and quantification were carried out as described in Bianchetti et al. (2018). Carotenoids (namely lycopene, β-carotene and lutein) were extracted and analyzed by high-pressure liquid chromatography (HPLC) with photodiode array detector (PDA). Carotenoid extraction was performed precisely as described by Bianchetti et al. (2018). Chromatography was carried out on an Agilent Technologies series 1100 HPLC system on a normal-phase column Phenomenex (Luna C18; 250 × 4.6 mm; 5 μm particle diameter) with a flow rate of 1 mL min^-1^ at 25°C. The mobile phase was a gradient of ethyl acetate (A) and acetonitrile:water 9:1 (v/v) (B): 0–4 min: 20% A; 4–30 min: 20–65% A; 30–35 min: 65% A; 35–40 min: 65–20% A. Eluted compounds were detected between 340 and 700 nm and quantified at 450 nm. The endogenous metabolite concentration was obtained by comparing the peak areas of the chromatograms with commercial standards.

### Fruit Surface Color Measurement

Fruit surface color was assessed with a using Konica Minolta CR-400 colorimeter, using the D65 illuminant and the L^∗^, a^∗^, b^∗^ space, and the data were processed to obtain hue and chroma values. Three measures were taken at the equator of each fruit and average values were calculated. The hue angle (in degrees) was calculated according to the following equations: hue = tan-1 (b^∗^/a^∗^) if a > 0 and 180 + tan-1 (b^∗^/a^∗^) if a < 0 (Ecarnot et al., 2013).

### Antioxidant Activity

Antioxidant activity was measured using the method of Trolox equivalent antioxidant capacity (TEAC). Frozen pericarp samples (approximately 200 mg FW) ground in liquid nitrogen were homogenized with 1 mL of 100 mM sodium acetate buffer (pH 5) and shaken for 30 min at 4°C. After centrifugation (4°C, 5000 *g*, 10 min), the supernatant was discarded, 0.5 mL of hexane was added to the pellet, and the suspension was kept shaking for 30 min at 4°C. After centrifugation (4°C, 5000 *g*, 10 min), the supernatant was collected, and the same process was repeated twice. The lipophilic antioxidant extract was concentrated and suspended in 150 μL of hexane. Absorbance was read at 734 nm after 2 h of incubation under darkness. The activity of the extract was determined by the deactivation of 2,2′-azino-bis(3-ethylbenzothiazoline-6-sulfonic acid (ABTS^+^) compared to a standard curve of 6-hydroxy-2,5,7,8-tetramethylchroman-2-carboxylic acid (Trolox).

### Auxin Measurements

Endogenous levels of indole acetic acid (IAA) were determined by gas chromatography-tandem mass spectrometry-selecting ion monitoring (GC-MS-SIM) as described by Santana-Vieira et al. (2016). Frozen pericarp samples (approximately 100 mg FW) were ground in liquid nitrogen and homogenized with 1 mL of isopropanol:acetic acid (95:5, v/v). Precisely 0.5 μg [^13^C_6_]-IAA (Cambridge Isotopes, Inc.) was added to each sample as internal standards. Samples were incubated at 4°C for approximately 2 h. After centrifugation (4°C, 16.000 *g*, 20 min), the supernatant was collected, and 100 μL of ultrapure water and 500 μl of ethyl acetate were added. After centrifugation (4°C, 16.000 *g*, 5 min) the supernatant was collected, and this step was repeated. The extract was completely vacuum dried and suspended in 50 μL methanol followed by a 30-min derivatization step at room temperature using 40 μL (trimethylsilyl)diazomethane.

The analysis was performed with a gas chromatograph coupled to a mass spectrometer (model GCMS-QP2010 SE, Shimadzu) in selective ion monitoring mode. One microliter of each sample was automatically injected (model AOC-20i, Shimadzu) on splitless mode, using helium as the carrier gas at a flow rate of 4.5 mL min^-1^ through a fused-silica capillary column (30 m, 0.25 mm ID, 0.50-μm-thick internal film) DB-5 MS stationary phase in the following program: 2 min at 100°C, followed by gradients of 10°C min^-1^ to 140°C, 25°C min^-1^ to 160°C, 35°C min^-1^ to 250°C, 20°C min^-1^ to 270°C and 30°C min^-1^ to 300°C. The injector temperature was 250°C, and the following MS operating parameters were used: ionization voltage, 70 eV (electron impact ionization); ion source temperature, 230°C; and interface temperature, 260°C. Ions with a mass ratio/charge (m/z) of 130 and 189 (corresponding to endogenous IAA) and 136 and 195 (corresponding to [_13_C^6^]-IAA) were monitored. Endogenous concentrations were calculated based on extracted chromatograms at m/z 130 and 136.

### Ethylene Emission

Ethylene emission was analyzed by gas chromatography with a flame-ionization detector (GC-FID) as described in Melo et al. (2016). Intact tomato fruits (typically 4 individuals) were enclosed in a sealed transparent tube for 1 h under specific experimental conditions. After incubation, 9-mL gas samples were collected from tubes and injected into a glass vial headspace previously flushed with ethylene-free air (1 L min^-1^) for 1 min. At least three 1-mL aliquots of each sample were injected in a headspace coupled to a Trace GC Ultra gas chromatography (Thermo Electron) fitted with a flame ionization detector (GC-FID) using an RT-alumina Plot column (Restek Corporation). Nitrogen was used as the carrier gas at a flow rate of 3 mL min^-1^, and commercial standard mixtures of ethylene were used for the calibration curves. Column, injector and detector temperatures were 34, 250, and 250°C, respectively.

### 1-Aminocyclopropane-1-Carboxylic Acid (ACC) Measurement

ACC was extracted and subsequently quantified as described by Bulens et al. (2011). Frozen pericarp samples (approximately 1 g FW) were ground in liquid nitrogen and homogenized with 4 mL of a 5% (w/v) sulfosalicylic acid aqueous solution. Extracts were shaken for 30 min at 4°C at 180 rpm in the dark. The supernatant was collected after centrifugation at 4°C, 5000 *g*, for 10 min. The reactions were performed by adding 1.4 mL of extract to a reaction mixture composed of 0.4 mL of 10 mM HgCl_2_ and 0.2 mL of a 2:1 (v/v) solution of NaOCl 5%:NaOH 6 M. The final product of this reaction, ethylene, was measured by GC-FID as described above.

### ACC Oxidase (ACO) Activity

ACO extraction and activity assay were performed according to Bulens et al. (2011). Frozen pericarp samples (approximately 100 mg FW) were ground in liquid nitrogen and homogenized with extraction buffer composed of 300 mM Tris-HCl (pH 8.0), 15 mg mL^-1^ insoluble polyvinylpolypyrrolidone (PVPP), 10% (v/v) glycerol and 30 mM ascorbic acid. After centrifugation (4°C, 20000 *g*, 20 min), 200 μL of the supernatant was added to 1.8 mL of reaction medium composed of 100 mM Tris-HCl (pH 8.0), 10% (v/v) glycerol, 30 mM ascorbic acid, 100 μM FeSO_4_, 50 mM NaHCO_3_, 5 mM DTT and 2 mM ACC. ACO activity was estimated by measuring the ability of the extract to convert exogenous ACC to ethylene after incubation at 30°C for 60 min. The ethylene formed during the reactions was measured by GC-FID as described above.

### Quantitative GUS Activity Assay

GUS activity was assayed according to Melo et al. (2016). Frozen pericarp samples (approximately 500 mg FW) were ground in liquid nitrogen and homogenized in 1 mL extraction buffer composed of 50 mM Hepes-KOH (pH 7.0), 5 mM DTT and 0.5% (w/v) PVP. After centrifugation (4°C, 20.000 *g*, 20 min), 200 μL aliquots of the supernatant were mixed with 200 μL of an assay buffer composed of 50 mM HEPES-KOH (pH 7.0), 5 mM DTT, 10 mM EDTA and 2 mM 4-methylumbelliferyl-β-D-glucuronide (MUG) and incubated at 37°C for 30 min. Subsequently, aliquots of 100 μL were taken from each tube and the reactions were stopped with 2.9 mL of 0.2 M Na_2_CO_3_ (pH 9.5). Fluorescence was measured using 365 nm excitation and 460 nm emission wavelength (5 nm bandwidth) by using a spectrofluorometer (LS55, Perkin Elmer). The same instrument settings were maintained throughout the experiments.

### Gene Promoter Analyses

Promoter sequences were retrieved from Sol Genomics Network^1^ and analyzed using PlantPAN 2.0 platform^2^ (Chow et al., 2016) to identify the regulatory motifs. Fragments of 3 kb upstream from the initial codon ATG were analyzed for the presence of PBE-box (CACATG), G-box (CACGTG), CA-hybrid (GACGTA) and CG-hybrid (GACGTG) motifs, which are recognized by HY5 and/or PIFs (Martínez-García et al., 2000; Song et al., 2008).

### RNA Isolation and Quantitative RT-PCR Analyses

Total RNA extraction was performed using ReliaPrep^TM^ RNA Tissue Miniprep System (Promega) according to manufacturer’s instructions for fibrous tissues. Total RNA and integrity of samples were determined using spectrophotometer and 1% (w/v) agarose gel. Only RNA samples with 260/280 and 260/230 ratio values within 1.8–2.2 were used for the subsequent steps. Approximately 1 μg of total RNA was treated with DNase (DNase I Amplification Grade, Thermo Fisher Scientific) for 30 min at room temperature and complementary DNA (cDNA) was synthesized using SuperScript^®^ IV Reverse Transcriptase kit (Thermo Fisher Scientific) according to manufacturer’s instructions. Only cDNA samples free of DNA contamination were used in the subsequent steps.

Quantitative reverse-transcriptase PCR (qPCR) reactions were performed using the StepOnePlus^TM^ Real-Time PCR System (Applied Biosystems) using 10 μl mix reaction composed of 5 μL Power SYBR green 2X (Thermo Fisher Scientific), 2 μL cDNA sample and 200 nM of forward and 200 nM of reverse primer. The amplification program consisted of 10 min initial step at 95°C, followed by 40 cycles with 15 s 95°C, 30 s 55/60°C and 30 s 72°C. Melting curve was analyzed to detect unspecific amplifications and primer dimerization. The primer sequences used in this study are listed in **Supplementary Table 1**. Fluorescence data were analyzed using LingReg PCR software, and expression values were normalized against mean values of two references genes: *Sl-EXPRESSED* and *Sl-CAC*, which have been already successfully used to normalize data from fruit development and ripening experiments (Expósito-Rodríguez et al., 2008; Bianchetti et al., 2018).

### Statistical Analysis

Two-way analysis of variance (ANOVA) was performed to determine effects of genotype, light treatment and their interactions, using JMP statistical software package (14th edition)^3^. One-way ANOVA with Tukey’s test or Student’s *t*-test was used to discriminate means of samples within and between genotypes, respectively. Comparisons with *P* < 0.05 were considered statistically significant. Carotenoid-related data were also compared via principal component analysis (PCA) using JMP statistical software package.

## Results

### Light Treatment and Loss of *Sl-DET1/HP2* Function Promote Fruit Carotenoid Biosynthesis

The impacts of *Sl-DET1*/*HP2* knockout or knockdown on tomato fruit carotenogenesis have been exclusively investigated in fruits ripening on-the-vine under greenhouse conditions (Davuluri et al., 2004; Kolotilin et al., 2007; Azari et al., 2010a; Enfissi et al., 2010; Sestari et al., 2014). However, after reaching the MG stage, tomato fruits are also able to ripen off-the-vine (i.e., isolated from the plant), a frequent commercial practice in harvesting tomato fruit for human consumption (Sorrequieta et al., 2013).

**FIGURE 1:**
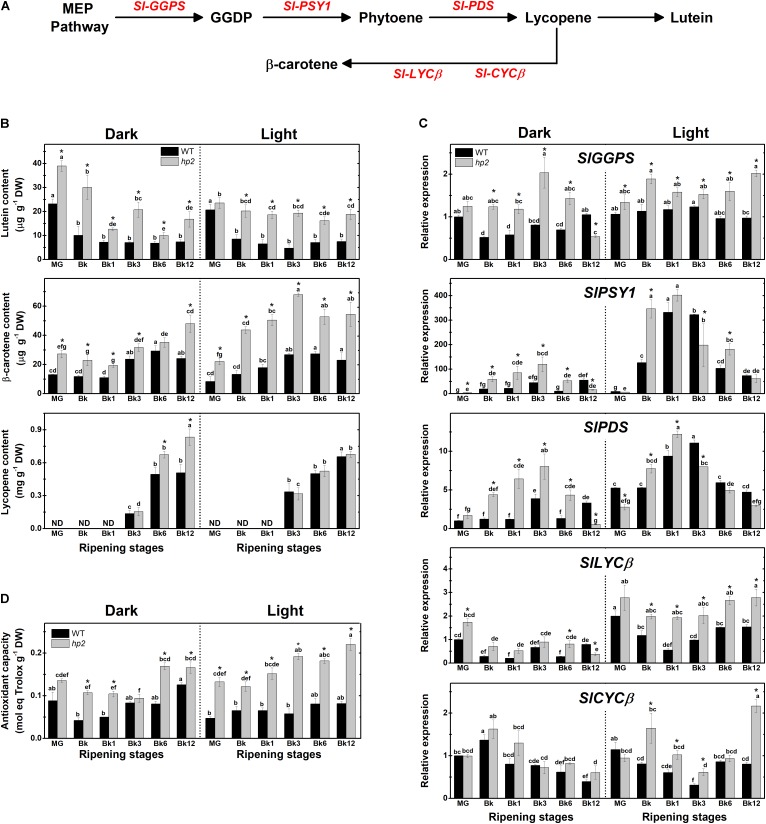
Loss of *Sl-DET1/HP2* function promotes tomato fruit carotenoid biosynthesis and antioxidant capacity in both dark- and light-ripened fruits. Wild-type (WT) and *high pigment-2* (*hp2*) fruits harvested at mature green (MG) stage were left to ripen under constant light (L) or dark (D) conditions. Pericarp samples were harvested at MG (2 days after the beginning of treatment), breaker (Bk), Bk1 (1 day after Bk), Bk3, Bk6, and Bk12 stages. **(A)** Schematic representation of carotenoid biosynthetic pathway in tomato. Intermediate reactions are omitted. **(B)** Lutein, β-carotene and lycopene content in pericarp tissues. **(C)** Relative mRNA levels of carotenoid biosynthesis genes. Mean relative expression was normalized against wild-type (WT) samples at mature green (MG) stage under dark conditions. **(D)** Trolox equivalent antioxidant capacity (TEAC) content in lipophilic extracts. Data are means (±SE) of at least three biological replicates. Different letters indicate statistically significant differences (Tukey’s test, *p* < 0.05) within each genotype. Asterisks indicate statistically significant differences (Student’s *t*-test, *p* < 0.05) between genotypes. MEP, Methylerythritol 4-phosphate; GGDP, Geranylgeranyl diphosphate; GGPS, GGDP synthase; PSY, Phytoene synthase; PDS, Phytoene desaturase; LCYβ, Chloroplast-specific β-lycopene cyclase; CYCβ, Chromoplast-specific β-lycopene cyclase.

Here, we demonstrated that the loss of *Sl-DET1*/*HP2* function promotes carotenogenesis even when tomato ripening occurs separated from the plant under either light or absolute dark conditions (**Figure 1** and **Supplementary Figure 1**). Two-way ANOVA showed that both the *hp2* mutation and the light treatment had a significant (*P* < 0.05) effect on carotenoid biosynthesis and accumulation (**Supplementary Table 2**). In both light- and dark-treated fruits, lutein and β-carotene levels were significantly higher in *hp2* than in the WT at virtually all sampling stages (**Figure 1B**). Moreover, lycopene levels of dark-treated *hp2* fruits were higher than the WT at the final stages of ripening (i.e., Bk6 and Bk12). In agreement, the genes encoding key carotenoid biosynthesis-related enzymes such as geranylgeranyl diphosphate synthase (GGPS), phytoene synthase 1 (PSY1) and phytoene desaturase (PDS) were strongly up-regulated during the climacteric phase (i.e., Bk to Bk6) in both light- and dark-treated *hp2* fruits compared with WT counterparts (**Figure 1C** and **Supplementary Figure 1**). Overall, *Sl-GGPS*, *Sl-PSY1* and *Sl-PDS* transcripts were less abundant in fruits maintained under dark than under light conditions, and this dark-induced reduction in mRNA levels was less marked in the *hp2* mutant compared to the WT (**Figure 1C**). Genes encoding the chloroplast- and chromoplast-specific β-lycopene cyclases (LYCβ and CYCβ, respectively) were also up-regulated in *hp2* fruits compared to the WT, particularly when ripening occurred under light conditions. Among the carotenoid biosynthesis-related genes differently expressed in *hp2* fruits, the additive effect of light treatment and loss of *Sl-DET1/HP2* function was particularly observed at the Bk, Bk1 and Bk12 stages (**Supplementary Figure 1**). Interestingly, lycopene levels were slightly higher in *hp2* fruits ripened in the dark than in light-treated ones (**Figure 1B**), which is very likely due to the accumulation of this carotenoid because the opposite pattern was observed for the transcript levels of *Sl-PSY1* and *Sl-PDS*, *i.e.*, higher mRNA levels in the light than in the dark conditions (**Figure 1C**).

In line with the increment in carotenoid content observed in *hp2* fruits, lipophilic extracts obtained from either dark- or light-incubated fruits of the mutant exhibited higher values of antioxidant capacity than the WT counterparts, a response intensified under light conditions (**Figure 1D**). The influence of the *hp2* mutation on lycopene, β-carotene and antioxidant capacity was moderated by the light treatment, as indicated by a significant genotype x light treatment interaction (*P* < 0.0001, **Supplementary Table 2**). Moreover, when PCA was performed with carotenoid data, the model explained 62.2% of the data variance for these conditions, displaying *hp2* samples separated from WT independently of the developmental stage or light condition, and a strong positive correlation between the changes in mRNA levels of genes encoding carotenoid biosynthetic genes with the fruit carotenoid composition and antioxidant capacity was confirmed (**Supplementary Figure 2**).

At MG, *hp2* fruits displayed a distinctive dark-green coloration, increased chlorophyll levels and higher color saturation (chroma, which is indicative of color intensity) compared to the WT (**Supplementary Figure 3**). In line with the higher content of pigments in *hp2* than in WT fruits, an overall trend of higher values of fruit color intensity was observed in the mutant fruits during ripening (Bk to Bk12) regardless of the light conditions (**Supplementary Figure 3**).

As dark-incubated *hp2* fruits showed carotenoid levels and lipophilic antioxidant capacity higher than dark- or even light-treated WT fruits, this mutation seems to represent a valid strategy to promote fruit nutritional quality even when the light stimulus is not present during fruit ripening.

### Light-Hypersensitivity Influences Tomato Fruit Ripening

**FIGURE 2:**
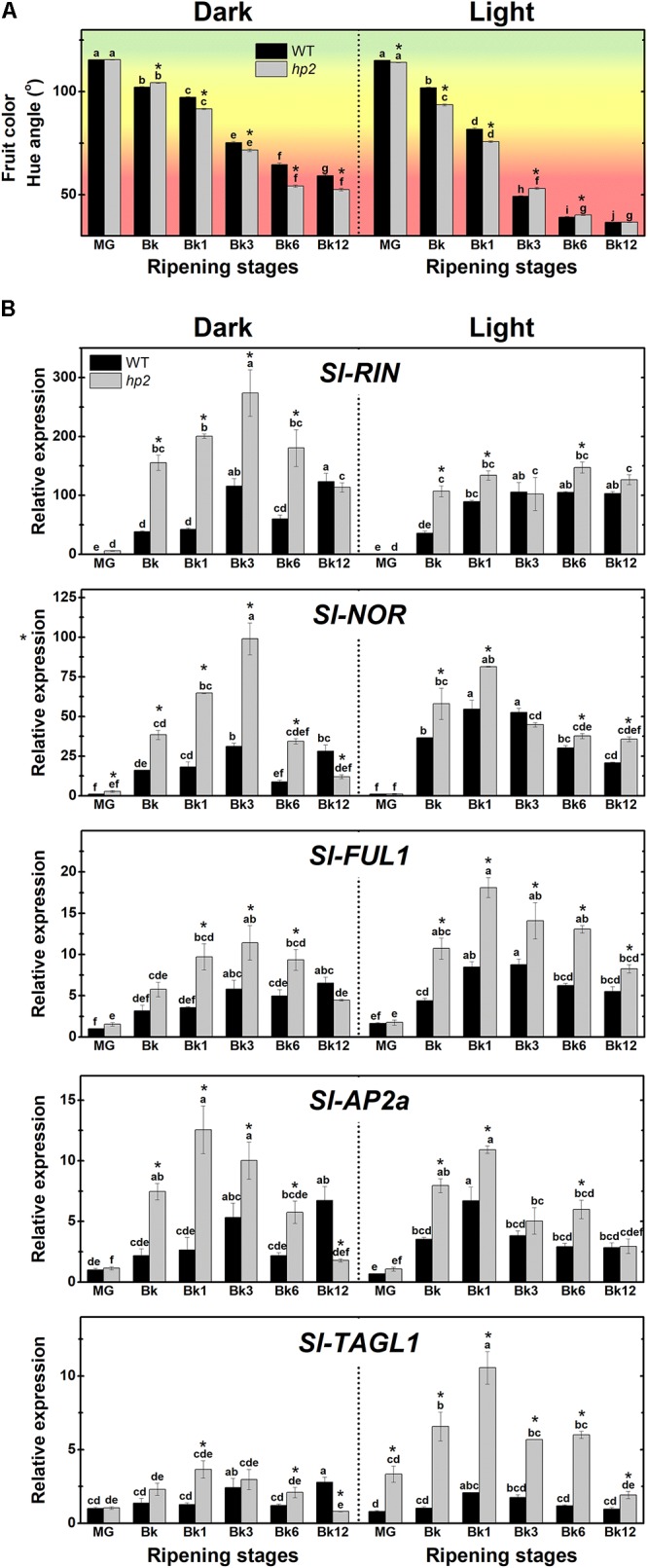
Light signaling influences tomato fruit ripening. Treatment details as described in **Figure 1**. **(A)** Ripening-related changes in fruit color (Hue angle). **(B)** Transcript abundance of ripening regulator genes in dark- and light ripened fruits. Mean relative expression was normalized against wild-type (WT) samples at mature green (MG) stage under dark conditions. Data are means (±SE) of at least three biological replicates. Different letters indicate statistically significant differences (Tukey’s test, *p* < 0.05) within each genotype. Asterisks indicate statistically significant differences (Student’s *t*-test, *p* < 0.05) between genotypes. *hp2*, *high pigment-2*; Bk, Breaker; RIN, ripening inhibitor; NOR, non-ripening; FUL1, fruitfull1; AP2a, apetala2a; TAGL1, tomato agamous-like1.

To investigate whether the loss of *Sl-DET1/HP2* function impacts tomato fruit ripening initiation and progression, we first monitored the ripening-associated changes in fruit color in both the *hp2* and WT genotypes (**Figure 2A**). Hue angle values revealed that light-incubated fruits acquired the distinctive red coloration faster and more intensively than those kept under complete darkness. Moreover, the ripening-associated fruit color transition occurred slightly faster in *hp2* than in WT fruits, particularly under dark conditions (**Figure 2A**).

In line with these results, mRNA levels of genes encoding the master regulators of ripening RIPENING INHIBITOR (RIN), NON-RIPENING (NOR), FRUITFULL1 (FUL1), APETALA2a (AP2a) and TOMATO AGAMOUS-LIKE1 (TAGL1) were significantly higher in *hp2* than in WT fruits ripening either under light or dark conditions (**Figure 2B**). Overall, the impact of the loss of *Sl-DET1/HP2* function on the transcript abundance of these ripening-associated genes was influenced by the light treatment, as indicated by a significant genotype x light treatment interaction (*P* < 0.05, **Supplementary Table 2**). Therefore, a positive correlation was observed between the up-regulation of the master regulators of ripening and the carotenoid overaccumulation observed in *hp2* ripening fruits. The promotive impact of the loss of *Sl-DET1*/*HP2* function on the expression of master regulators of ripening may also be linked to the slightly faster fruit color transition observed in the mutant compared to the WT under dark conditions (**Figure 2A**). Accordingly, HY5- and/or PIF-binding motifs were identified in the promoter regions of all five master regulators of ripening genes analyzed (**Supplementary Figure 4**).

### Loss of *Sl-DET1/HP2* Function Alters Ethylene Biosynthesis, Signaling and Responsiveness During Tomato Ripening

**FIGURE 3:**
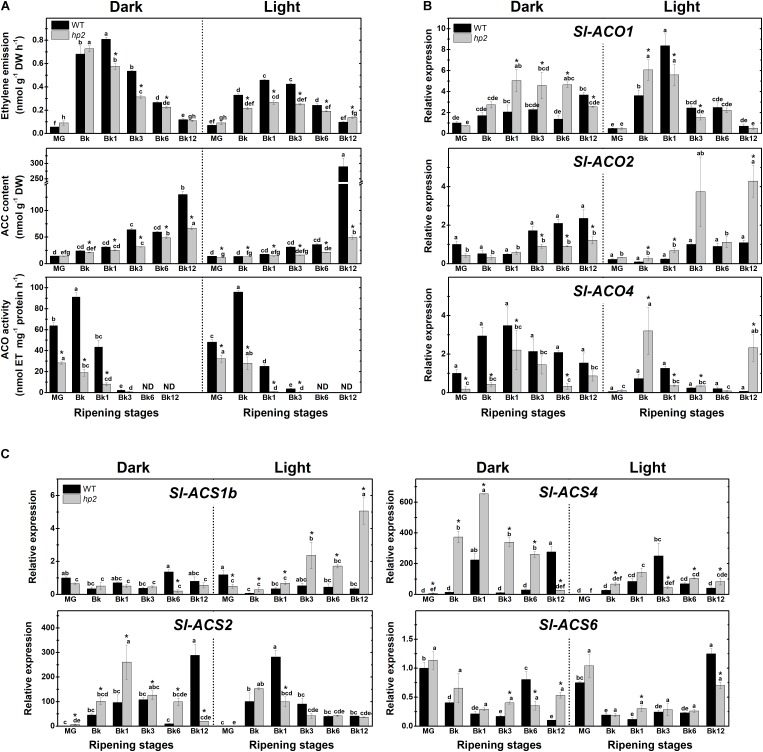
Light-hypersensitivity represses ethylene metabolism in ripening tomato fruits. Treatment details as described in **Figure 1**. **(A)** Ethylene emission, 1-aminocyclopropane-1-carboxylic acid (ACC) content, *in vitro* ACC oxidase (ACO) activity. **(B)** Relative mRNA levels of tomato genes encoding ACO. **(C)** Relative mRNA levels of tomato genes encoding ACC synthase (ACS). Mean relative expression was normalized against wild-type (WT) samples at mature green (MG) stage under dark conditions. Data are means (±SE) of at least three biological replicates. Different letters indicate statistically significant differences (Tukey’s test, *p* < 0.05) within each genotype. Asterisks indicate statistically significant differences (Student’s *t*-test, *p* < 0.05) between genotypes. *hp2*, *high pigment-2*; Bk, Breaker.

To gain insight into the potential influence of light treatment and the loss of *Sl-DET1*/*HP2* function on fruit ethylene metabolism, we next monitored ethylene emission, 1-aminocyclopropane-1-carboxylic acid (ACC) content, ACC oxidase (ACO) activity and transcript abundance of ethylene biosynthetic genes in WT and *hp2* ripening fruits. In both genotypes and light conditions, the highest values of ethylene emission were detected from Bk to Bk3 (**Figure 3A**). Also, ACC accumulated at the end of the ripening (Bk12) in all conditions analyzed, which was associated with a drastic reduction in ACO activity from BK stage onward (**Figure 3A**). Compared to the WT, *hp2* fruits exhibited significantly lower ethylene emission rates, ACC content and ACO activity regardless of the light treatment. In both genotypes, climacteric ethylene emission was significantly lower under light than under dark conditions (**Figure 3A**).

To investigate whether these light-induced alterations in ethylene emission were associated with changes in the transcriptional profile of ethylene biosynthetic genes, the mRNA levels of all ACS- and ACO-encoding genes responsible for the climacteric ethylene burst in ripening tomato fruits were profiled. Overall, the influence of light exposure or the *hp2* mutation on the transcript abundance of these genes was highly variable, greatly varying depending on the gene analyzed or the ripening stage (**Figures 3B,C**). Therefore, no clear correlation was observed between the transcriptional regulation of tomato ACS- and ACO-encoding genes (**Figures 3B,C**) and the reduced ethylene biosynthesis (**Figure 3A**) observed in light compared to the dark treatment or in *hp2* compared to the WT genotype. Together, these findings indicate that light exposure and the *hp2* mutation, either combined or isolated, can cause an overall down-regulation in tomato ethylene biosynthesis, which is associated with complex changes in the transcriptional profile of *ACS* and *ACO* genes.

**FIGURE 4:**
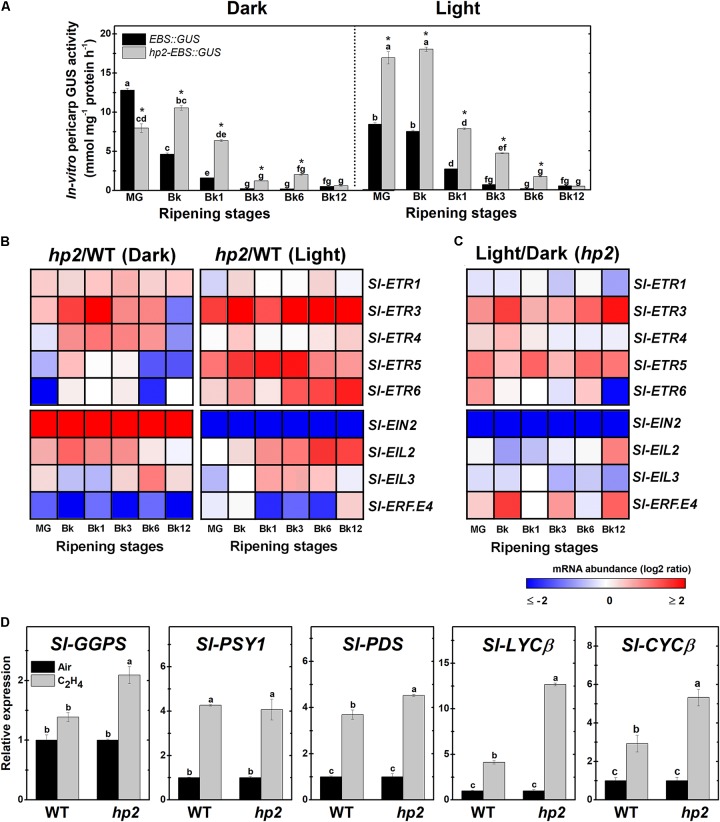
Loss of *Sl-DET1/HP2* function promotes ethylene tissue sensitivity and signaling output. Treatment details as described in **Figure 1**. **(A)** *In vitro* GUS activity assayed in wild-type (WT) and *high pigment-2* (*hp2*) fruits carrying the ethylene-responsive promoter *EBS* fused to the GUS reporter protein (*EBS::GUS* and *hp2*-*EBS::GUS*). **(B)** Heatmap representation of the differences in relative mRNA levels of ethylene perception and signaling-related genes between the WT and *hp2* fruits ripened under light or dark conditions. **(C)** Heatmap representation of the differences in relative mRNA levels of ethylene perception and signaling-related genes between light and dark samples of *hp2* fruits at each sampling time. The relative transcript values are presented in **Supplementary Figure 5**. **(D)** Relative mRNA levels of tomato genes encoding carotenoid biosynthetic enzymes in WT and *hp2* fruits treated with 50 ppm ethylene for 6 h. Data are means (±SE) of at least three biological replicates. Different letters indicate statistically significant differences (Tukey’s test, *p* < 0.05) within each genotype (in **A**) or among all data (in **C**). In A, asterisks indicate statistically significant differences (Student’s *t*-test, *p* < 0.05) between genotypes. MG, mature green; Bk, Breaker; ETR, ethylene response; EIN, ethylene insensitive; EIL, ethylene insensitive 3-like; ERF, ethylene response factor; GGPS, geranylgeranyl diphosphate synthase; PSY, phytoene synthase; PDS, phytoene desaturase; LCYβ, chloroplast-specific β-lycopene cyclase; CYCβ, chromoplast-specific β-lycopene cyclase.

Based on these findings, we further investigated whether light hypersensitivity alters ethylene signaling in ripening tomato fruits. First, the ethylene signaling output was determined by monitoring the activity of the reporter protein GUS expressed under the control of the *EBS* ethylene-responsive promoter in *EBS::GUS* and *hp2-EBS::GUS* genotypes. Whether under light or dark conditions, the highest GUS activity values in both genotypes coincided with the climacteric burst of ethylene production (**Figure 4A**). However, the loss of *Sl-DET1/HP2* function resulted in higher *EBS* promoter activation, and this phenomenon was clearly intensified by the presence of light (**Figure 4A**).

The altered ethylene signaling output observed in *hp2* fruits was associated with marked differences in the transcript abundance of genes involved in ethylene perception and signaling (**Figure 4B** and **Supplementary Figure 5**). *ETHYLENE RESPONSE 3* (*Sl-ETR3*), one of the tomato ethylene receptor genes most highly expressed during ripening initiation (Liu M. et al., 2015), was strongly up-regulated in *hp2* compared to the WT regardless of the light conditions. To a certain extent, a similar trend was also observed for some other ETR genes, including *Sl-ETR4*, *Sl-ETR5* and *Sl-ETR6.*

The mRNA levels of *ETHYLENE INSENSITIVE 2* (*Sl-EIN2*), which encodes a key component in the ethylene signaling cascade, was differentially affected by the *hp2* mutation depending on the light conditions, being more greatly expressed in *hp2* than in WT fruits in the dark and displaying the opposite pattern under light conditions (**Figure 4B** and **Supplementary Figure 5**). Transcript levels of both primary (ETHYLENE INSENSITIVE 3-LIKE, EIL) and secondary (ETHYLENE RESPONSE FACTOR, ERF) ethylene-related transcription factors were also altered in *hp2* fruits compared to the WT. Both *Sl-EIL2* and *Sl-EIL3* were more abundantly expressed in *hp2* than in the WT fruits whereas the opposite was observed for the *Sl-ERF.E4*, which encodes a repressor of tomato carotenogenesis (Lee et al., 2012). The additive effect of light treatment and loss of *Sl-DET1/HP2* function was particularly observed for *Sl-ETR3*, *Sl-ETR5*, and *Sl-EIN2* (**Figure 4C**).

All tomato ethylene receptor and signaling-related genes analyzed, except *Sl-ETR3* and *Sl-EIN2*, displayed HY5 and PIF-binding motifs within their 3-kb promoter sequences (**Supplementary Figure 6**). Interestingly, *Sl-EIN2* was the only ethylene-related gene that was differentially affected by the loss of *Sl-DET1/HP2* function depending on the light conditions (**Figure 4B**).

To further investigate the relationship between ethylene responsiveness and carotenoid biosynthesis, carotenoid biosynthetic genes were profiled in both WT and *hp2* fruits at MG stage exposed to a short-term (6h) treatment with exogenous ethylene (**Figure 4D**). All genes profiled, except for *Sl-GGPS*, were significantly up-regulated in WT fruits, thereby validating the efficacy of the ethylene treatment and confirming the positive influence of this hormone on the transcriptional regulation of the carotenoid pathway in tomato fruits. Comparatively, the ethylene-induced up-regulation of genes such as *Sl-GGPS*, *Sl-PDS*, and particularly *Sl-LYCβ* and *Sl-CYCβ*, was significantly more pronounced in *hp2* than in the WT fruits, which corroborates the hypothesis that the increased responsiveness of *hp2* fruits to ethylene may be associated with the overaccumulation of carotenoids in this mutant.

### Light-Hypersensitivity Promotes Auxin Responsiveness in Tomato Fruits

In concert with ethylene, auxin is also part of the regulatory network controlling tomato fruit ripening and carotenoid synthesis (Su et al., 2015). To evaluate whether the carotenoid overaccumulation and altered ethylene signaling observed in *hp2* fruits are associated with changes in auxin levels and signaling, we next compared the endogenous IAA content, *DR5* promoter activation and transcriptional profile of genes encoding auxin-related signaling elements in WT and *hp2* ripening fruits.

**FIGURE 5:**
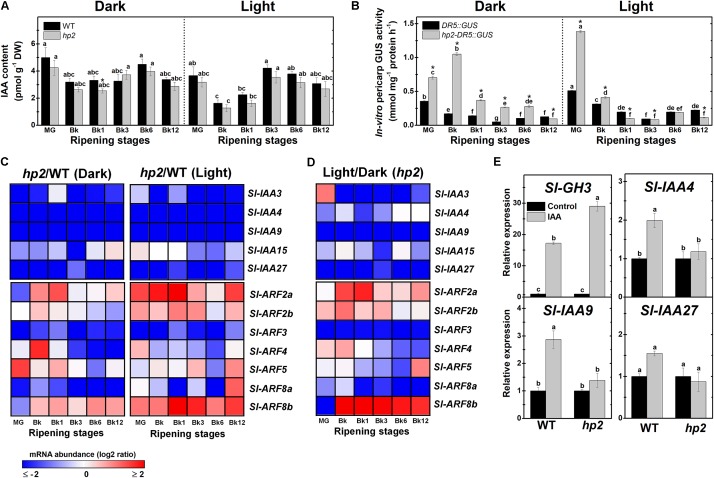
Light-hypersensitivity promotes auxin sensitivity and signaling without altering endogenous IAA levels. Treatment details as described in **Figure 1**. **(A)** Endogenous indole-3-acetic acid (IAA) levels in wild-type (WT) and *high pigment-2* (*hp2*) fruits. **(B)** *In vitro* GUS activity assayed in WT and *hp2* fruits carrying the auxin-responsive promoter *DR5* fused to the GUS reporter protein (*DR5::GUS* and *hp2*-*DR5::GUS*). **(C)** Heatmap representation of the differences in mRNA levels of auxin signaling genes between the WT and *hp2* fruits ripened under light or dark conditions. **(D)** Heatmap representing differences in mRNA levels of auxin signaling genes between light and dark samples of *hp2* fruits at each sampling time. The relative transcript values are presented in **Supplementary Figures 7**, **8**. **(E)** Relative mRNA levels of auxin-responsive genes in WT and *hp2* fruits treated with 100 μM IAA for 6 h. Data are means (±SE) of at least three biological replicates. Different letters indicate statistically significant differences (Tukey’s test, *p* < 0.05) within each genotype (in **A,B**) or among all data (in **E**). In **(A,B)**, asterisks indicate statistically significant differences (Student’s *t*-test, *p* < 0.05) between genotypes. MG, mature green; Bk, Breaker; Aux/IAA, auxin/indole-3-acetic acid; ARF, auxin response factor.

Endogenous IAA levels were remarkably similar in WT and *hp2* ripening fruits (**Figure 5A**). In contrast, the activity of the reporter protein GUS expressed under the control of the auxin-responsive *DR5* promoter was considerably higher in either light or dark-incubated fruits of *hp2-DR5::GUS* compared to the *DR5::GUS* (**Figure 5B**). In both genotypes, a progressive reduction in auxin signaling output, as indicated by the *DR5* promoter activation, was observed during fruit ripening. Auxin signaling output remained higher in *hp2-DR5::GUS* than in the *DR5::GUS* fruits from MG to Bk6 and from MG to Bk stage in dark- and light-incubated fruits, respectively.

As the higher auxin signaling output detected in *hp2* fruits were not associated with marked differences in endogenous IAA content between the genotypes (**Figures 5A,B**), it seems plausible to suggest that *hp2* fruits display increased sensitivity to this hormone compared to the WT. Corroborating these findings, the *hp2* mutation was found to trigger marked changes in the transcriptional profile of genes encoding auxin-associated signaling proteins such as Aux/IAA and ARFs (**Figure 5C**).

Among the five *Aux/IAA* tomato genes closely associated with fruit ripening – i.e., *Sl-IAA3, Sl-IAA4, Sl-IAA9, Sl-IAA15* and *Sl-IAA27* (Audran-Delalande et al., 2012) – a dramatic reduction in *Sl-IAA3*, *Sl-IAA4, Sl-IAA9* and *Sl-IAA27* mRNA levels in *hp2* compared to WT fruits was observed (**Figure 5C** and **Supplementary Figure 7**). *Sl-IAA15* mRNA levels were also reduced in *hp2* compared to the WT at certain ripening stages. Therefore, regardless of the light conditions, an overall down-regulation of *Sl-IAA* genes was observed in *hp2* fruits compared to the WT. The repressor role of light in the expression of these *Aux/IAA* genes was supported by the additive effect of light treatment and loss of *Sl-DET1/HP2* function on the mRNA levels of all *Aux/IAA* genes analyzed (**Figure 5D**).

The marked impact of loss of *Sl-DET1*/*HP2* function on auxin signaling output and *Aux/IAA* mRNA levels, prompted us to investigate whether changes in light signaling cause significant alterations in the transcript abundance of seven *ARF* genes highly expressed in ripening tomato fruits, i.e., *Sl-ARF2a*, *Sl-ARF2b*, *Sl-ARF3*, *Sl-ARF4*, *Sl-ARF5*, *Sl-ARF8a* and *Sl-ARF8b*. Data showed that transcript levels of *Sl-ARF2a* and *Sl-ARF2b*, which are considered key convergence points of auxin and ethylene signaling and important promoters of tomato fruit ripening and carotenoid biosynthesis (Hao et al., 2015; Breitel et al., 2016), were higher in *hp2* than in WT fruits (**Figure 5C** and **Supplementary Figure 8**). Similarly, mRNA levels of *Sl-ARF8b*, a known activator of auxin-dependent gene transcription (Kumar et al., 2014), were considerably higher in *hp2* than in WT fruits. Conversely, transcript abundance of *Sl-ARF3*, a well-established repressor of auxin-dependent gene transcription (Zouine et al., 2014), was dramatically reduced in *hp2* than in WT fruits regardless of the light treatment (**Figure 5C**). An overall reduction in *Sl-ARF8a* mRNA levels was also detected in *hp2* fruits compared to the WT, particularly under dark conditions. In contrast, the impacts of the loss of *Sl-DET1*/*HP2* function on *Sl-ARF4* and *Sl-ARF5* mRNA levels were considerably more variable as these genes were either up- or down-regulated in *hp2* compared to the WT depending on the ripening stage considered (**Supplementary Table 2**, i.e., non-significant influence of the genotype and the genotype × light treatment interaction). The combined effect of light exposure and the *hp2* mutation was clearly observed for all tomato ARF genes analyzed (**Figure 5D**). In summary, among all light-triggered alterations in the transcriptional profile of *Sl-ARF* genes, *Sl-ARF3* mRNA levels were down-regulated in response to both light exposure and the loss of *Sl-DET1*/*HP2* function, with the opposite being observed for *Sl-ARF2a*, *Sl-ARF2b*, and *Sl-ARF8b*.

Finally, the relationship between light and auxin responsiveness in *hp2* was also investigated by comparing the impacts of auxin treatment on the transcript abundance of the auxin-responsive genes *Sl-GH3*, *Sl-IAA4*, *Sl-IAA9* and *Sl-IAA27* (**Figure 5E**). Although *Sl-GH3* was clearly up-regulated in both WT and *hp2* fruits, the auxin-triggered accumulation of transcripts of this gene was significantly higher in the mutant, which further indicates increased auxin sensitivity in *hp2* compared to WT fruits. Auxin treatment promoted *Sl-IAA4*, *Sl-IAA9* and *Sl-IAA27* transcript accumulation in WT fruits but failed to alter the expression of these genes in *hp2* fruits (**Figure 5E**). These results are in line with the detection of lower *Sl-IAA* mRNA levels in the *hp2* compared to the WT, although both genotypes displayed similar endogenous IAA levels throughout the ripening phase (**Figures 5A,C**).

## Discussion

Assumptions that light-hormonal crosstalk may be involved in controlling tomato fruit ripening and carotenoid metabolism have been formulated for a long time in the literature (Lieberman, 1979; Yang and Hoffman, 1984), while unequivocal genetic or physiological evidence supporting this hypothesis remained lacking. As a major regulator of numerous ripening-associated processes, ethylene was one of the first hormones investigated as part of the regulatory mechanisms behind the light-dependent regulation of fruit carotenoid biosynthesis (Alba et al., 2000).

In vegetative tissues, ethylene biosynthesis is highly regulated by light quality, intensity and duration. Overall, light perception via photoreceptors, such as PHYs and CRYs, inhibits ethylene emission (Corbineau et al., 1995; Vandenbussche et al., 2003; Pierik, 2004; Giliberto et al., 2005; Melo et al., 2016), ACC accumulation (Jiao et al., 1987; Melo et al., 2016), ACO activity (Melo et al., 2016) and *ACS* transcript levels (Khanna et al., 2007). Our data revealed that the negative influence of light on ethylene metabolism typically found in vegetative tissues is also observed in ripening tomato fruits as indicated by the light-triggered reduction in ACC content, ACO activity and ethylene emission, a response that was further intensified in fruits of the light-hyperresponsive *hp2* mutant. The main *ACS* and *ACO* family members expressed during ripening were either up- or down-regulated in response to light exposure or the loss of *Sl-DET1*/*HP2* function, suggesting that light-dependent down-regulation of the ethylene climacteric burst in tomato is linked to complex alterations in the transcript abundance of its biosynthetic genes. These findings contrast with the observation that PHY-mediated light perception in plant vegetative tissues is frequently associated with the modulation of ethylene biosynthesis via conspicuous changes in the *ACS* transcription (Rodrigues et al., 2014), as illustrated by the several 100-fold enhancements in *AtACS4* transcript abundance detected in *Arabidopsis* seedlings overexpressing *AtPIF5* (Khanna et al., 2007).

**FIGURE 6:**
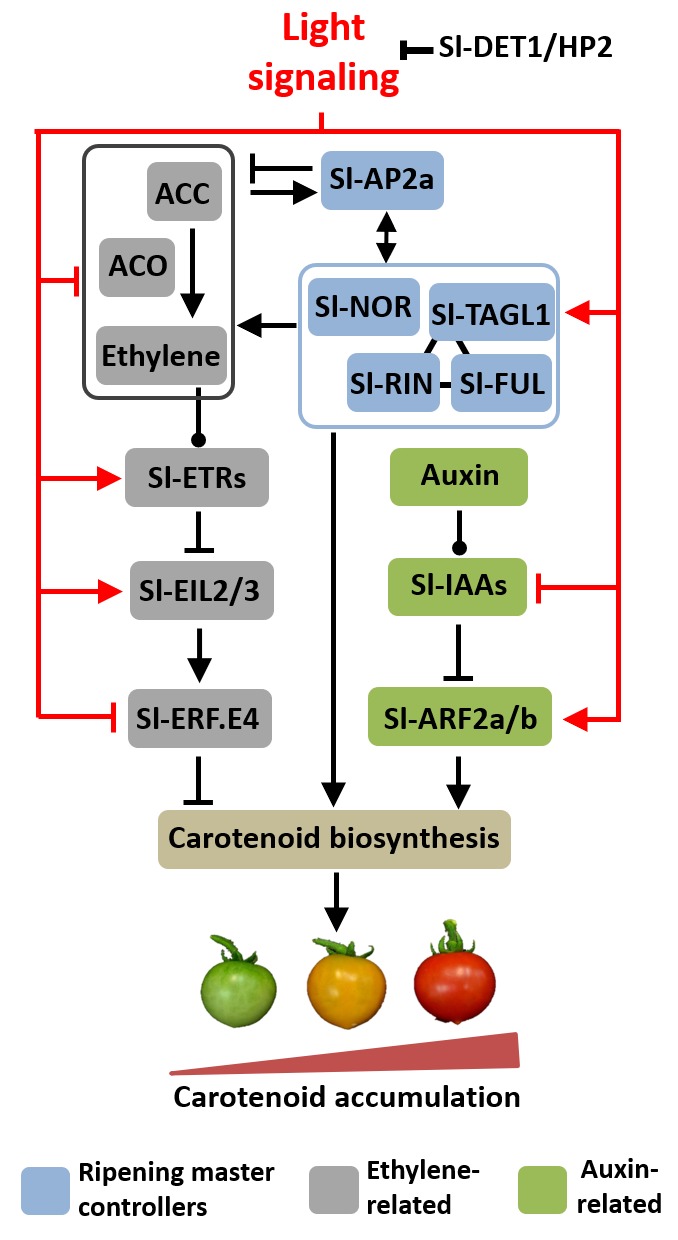
Proposed model for light-hormonal interaction controlling tomato fruit carotenoid biosynthesis. Light signaling promotes master regulators of ripening, which positively regulate carotenoid biosynthetic genes. Light also down-regulates ethylene metabolism and emission and reduces the expression of *ETHYLENE RESPONSE FACTOR E4* (*Sl-ERF.E4*), a key repressor of tomato fruit carotenogenesis. Moreover, light represses *AUXIN/INDOLE-3-ACETIC ACID* (*Sl-IAAs*) genes and up-regulates both *AUXIN RESPONSE FACTOR 2* paralogues (*SlARF2a/b*), consequently promoting most components of the tomato carotenoid biosynthetic route. Additionally, light signaling up-regulates genes encoding ethylene receptor (ETRs) and intermediate components in ethylene signaling cascade (EILs). The arrow- and bar-headed lines indicate stimulatory and inhibitory effects, respectively. The lines terminated by a circle describe more complex interactions. RIN, ripening inhibitor; NOR, non-ripening; FUL, fruitfull; AP2a, apetala2a, TAGL1, tomato agamous-like1; ETR, ethylene response; EIL, ethylene insensitive 3-like.

Ethylene biosynthesis in tomato fruits is tightly regulated by master regulators of ripening, stimulated by Sl-RIN, Sl-NOR, Sl-FUL, and Sl-TAGL1 (Itkin et al., 2009; Liu M. et al., 2015) and repressed by Sl-AP2a (Karlova et al., 2011). The up-regulation of all these ripening master regulators in *hp2* ripening fruits entails a complex interaction between the light signaling cascade and the regulatory cascade controlling ripening. Sl-AP2a acts as a negative regulator of tomato climacteric ethylene synthesis via a negative feedback loop (Karlova et al., 2011); therefore, the reduced ethylene production detected in *hp2* fruits may be associated with the up-regulation of *Sl-AP2a* in this mutant (**Figure 6**). In contrast, all ripening master regulators analyzed are well-established promoters of fruit carotenoid biosynthesis (Itkin et al., 2009; Martel et al., 2011; Liu L. et al., 2015); hence, their up-regulation in *hp2* ripening fruits is consistent with the over-accumulation of carotenoids in the mutant.

Besides altering ethylene biosynthesis, the loss of *Sl-DET1*/*HP2* function also impacted tomato fruit responsiveness to ethylene, a response associated with marked changes in ethylene receptors (ETRs) and downstream signaling transduction elements (EIN, EILs, ERFs). The receptor signaling model states that ETRs, including those involved in tomato ripening (i.e., Sl-ETR3 and Sl-ETR4), are negative regulators of ethylene responses (Kevany et al., 2007; Kamiyoshihara et al., 2012); consequently, reductions in the abundance of receptors promote tissue ethylene sensitivity (Tieman et al., 2000). However, information about the temporal fluctuations in ETR transcripts and protein levels during tomato ripening is controversial. Opposite temporal patterns between the mRNA and protein levels of Sl-ETR3 and Sl-ETR4 have been observed during tomato ripening, as the protein and transcript abundance of these receptors peak at the immature and ripening stages, respectively (Kevany et al., 2007). However, no significant alterations in ethylene receptor protein abundance were observed during tomato fruit ripening in a subsequent study (Kamiyoshihara et al., 2012). Therefore, on the one hand, the apparent contradiction between the up-regulation of *ETR* transcripts and the increased ethylene sensitivity detected in *hp2* fruits may be explained by the inverse pattern in ethylene receptor mRNA and protein levels already observed in tomato fruits (Kevany et al., 2007). On the other hand, if the *hp2*-triggered up-regulation of *Sl-ETR* expression results in increased receptor protein abundance, the increased ethylene sensitivity observed in the fruits of these mutants may be associated with a more complex alteration in ethylene perception and signaling cascade.

Acting downstream to ETR receptors, EIN2 plays a significant role in ethylene signaling by stabilizing EIN3/EIL transcription factors, which in turn will activate the transcription of multiple ethylene-responsive genes, including secondary transcription factors (i.e., ERFs) (Alonso, 1999). Many of these downstream signaling transduction elements are involved in photomorphogenic responses, sometimes acting as integrators of light and ethylene signaling during vegetative plant development (Zhong et al., 2009). In tomato, *Sl-EIN2*, *Sl-EIL* or *Sl-ERF.E4* suppression disturbed fruit ripening and, consequently, altered carotenoid metabolism (Tieman et al., 2001; Fu et al., 2005; Lee et al., 2012). As these genes were differentially expressed in *hp2* fruits compared to the WT, it seems that disturbances in light signaling can affect multiple steps in the ethylene transduction cascade, which may contribute to explain the altered ethylene responsiveness detected in this mutant. In this context, it is also worth mentioning that *Sl-ERF.E4* mRNA levels were severely reduced in *hp2* fruits and this ERF has been proposed as a major repressor of carotenoid synthesis in tomato, as revealed by the over-accumulation of this class of isoprenoid in fruits of *Sl-ERF.E4*-knockdown transgenic lines (Lee et al., 2012). Therefore, it seems that the increased ethylene responsiveness of *hp2* fruits may compensate for its reduced ethylene biosynthesis, which is supported by the comparatively higher expression of carotenoid biosynthetic genes in the mutant when both WT and *hp2* fruits were supplemented with the same concentration of ethylene.

Tomato fruit carotenogenesis is undeniably regulated by ethylene-related signaling components, but other plant hormones have also been increasingly implicated in controlling this metabolic pathway (Kumar et al., 2014; Liu L. et al., 2015). Auxins, for instance, have been demonstrated to counteract the promotive influence of ethylene on tomato fruit ripening and carotenogenesis (Pirrello et al., 2012; Su et al., 2015). Here, we provide several lines of evidence indicating that the loss of *Sl-DET1*/*HP2* function promotes auxin responsiveness in fruit tissues via changes in the transcript abundance of auxin signaling-related genes. The increased activation of *DR5* promoter in *hp2* fruits was not associated with significant differences in the endogenous IAA levels between the mutant and WT genotypes but instead was accompanied by the down-regulation of the *Sl-IAA* genes most greatly expressed in tomato fruits (i.e., *Sl-IAA3*, *Sl-IAA4*, *Sl-IAA9*, and *Sl-IAA27*).

Accordingly, functional characterization studies have revealed that the down-regulation of *Sl-IAA3*, *Sl-IAA9* or *Sl-IAA27* disturbs auxin responsiveness in tomato plants. Whereas *Sl-IAA3* knockdown resulted in lower auxin sensitivity, *Sl-IAA9*- or *Sl-IAA*27-silenced lines exhibited increased auxin responsiveness (Wang et al., 2005; Chaabouni et al., 2009; Bassa et al., 2012). Therefore, the progressive reduction in *DR5* promoter activity observed from the MG to Bk12 stages in both dark- and light-incubated fruits may be linked to the gradual increment in transcripts of the repressor of auxin responsiveness *Sl-IAA3* (Chaabouni et al., 2009), and the progressive reduction in transcripts of *Sl-IAA9* and *Sl-IAA*27, two positive regulators of tissue responsivity to auxins (Wang et al., 2005; Bassa et al., 2012). Among these tomato *Aux/IAA* genes, *Sl-IAA3* has been suggested to represent a crossroad of auxin and ethylene signaling in tomato, being highly regulated by both these hormones. Recent findings also indicate that Sl-IAA3 mediates the interplay between light and ethylene signaling, since dark- and light-grown *Sl-IAA3*-knockdown tomato seedlings exhibited marked differences in ethylene sensitivity (Chaabouni et al., 2009) and this tomato *Aux/IAA* gene was particularly up-regulated in ripening fruits of PHY-deficient tomato plants (Bianchetti et al., 2017). Therefore, it seems tempting to speculate that the light-dependent down-regulation of Sl-IAA3 may be associated with the increased responsivity to ethylene observed in *hp2* fruits.

Aux/IAA proteins are known to inhibit the activity of ARF, and ARFs can either act as transcriptional repressors or activators of auxin-responsive genes (Zouine et al., 2014). Hence, changes in ARF abundance also significantly impact plant tissue responsiveness to auxins (Sagar et al., 2013; Zouine et al., 2014; Hao et al., 2015). Accordingly, the increased auxin responsiveness observed in *hp2* fruits was associated with a marked down- and up-regulation of *Sl-ARF3* and *Sl-ARF8b*, respectively, as the former is a repressor of auxin-dependent gene transcription whereas the latter is an activator of auxin responses. In both cases, the impact of the *hp2* mutation was intensified by light exposure, which suggests that the light-dependent transcriptional regulation of these two *ARF*s may be associated with the increased auxin responsiveness observed in the *hp2* fruits.

The up-regulation of *Sl-ARF2a* and *Sl-ARF2b* caused by the loss of *Sl-DET1*/*HP2* function is also consistent with the proposed role suggested for these two *ARF*s on tomato fruit ripening and carotenogenesis (Hao et al., 2015; Breitel et al., 2016). Both *Sl-ARF2* paralogs are known to cooperate in promoting the expression of master controllers of ripening, such as Sl-RIN and Sl-NOR, stimulating ethylene biosynthesis and signaling and inducing carotenoid biosynthesis (Hao et al., 2015; Breitel et al., 2016). Therefore, the up-regulation of both *Sl-ARF2a* and *Sl-ARF2b* genes observed in light-incubated *hp2* fruits agrees with the higher expression of genes encoding master controllers of ripening and carotenoid biosynthetic enzymes detected in this light-hyperresponsive mutant.

Here, we put forward the hypothesis that light-triggered changes in auxin and ethylene responsiveness and signaling are associated with the overaccumulation of carotenoids in *hp2* fruits. In the proposed working model of light-hormonal crosstalk controlling tomato carotenogenesis (**Figure 6**), the positive and negative influence of light on ethylene biosynthesis and signaling, respectively, are supported by both genetic (i.e., *hp2* mutation versus WT genotype) and physiological evidence (i.e., light versus dark treatment). The assumption that light modulates auxin signaling is corroborated by the marked down-regulation of *Aux/IAA* tomato genes and altered *ARF* expression profile in *hp2* fruits compared to the WT. The two *ARF* genes most closely associated with tomato fruit ripening and carotenogenesis (i.e., *Sl-ARF2a* and *Sl-ARF2b*) and the genes encoding the master regulators of ripening (e.g., Sl-RIN, Sl-NOR, Sl-FUL1, Sl-AP2a) were up-regulated, whereas *Sl-ERF.E4*, a repressor of tomato fruit carotenogenesis, was repressed in *hp2* fruits compared to WT counterparts. All these changes in the central ripening-related regulatory modules are consistent with the increased transcript abundance of carotenoid biosynthetic genes (e.g., *Sl-GGPS*, *Sl-PSY1*, *Sl-PDS*, *Sl-LYCβ* and Sl*-LYCβ*) and the over-accumulation of carotenoids typically observed in the *hp2* mutant.

Although DET1 has long been identified as a major repressor of light signaling in plants (Chory et al., 1989), the molecular mechanisms responsible for its action on photomorphogenesis remain not yet fully characterized. However, accumulating evidence indicates that DET1 may interfere with multiple steps of the light signaling cascades. In Arabidopsis, DET1 interacts with DDB1 and COP10 to form the CDD complex, which physically associates with CUL4, giving rise to an E3 ligase that promotes the proteolytic degradation of photomorphogenesis-promoting factors, including HY5 (Yanagawa et al., 2004). DET1 has also been shown to positively and negatively regulate the accumulation of PIF and DELLA proteins, respectively (Dong et al., 2014; Li et al., 2015), which partially explains how DET1 represses *Arabidopsis* photomorphogenesis in darkness. Data also implicates DET1 action in chromatin remodeling (Benvenuto et al., 2002) and as a transcriptional co-repressor of key regulators of the circadian clock genes (Lau et al., 2011). Therefore, multiple mechanisms may be involved in the Sl-DET1/HP2-mediated regulation of ethylene and auxin pathways in ripening tomato fruits, including its influence on balancing HY5 and PIF protein abundance, possible global alterations in gene expression via chromatin remodeling, and its potential action as a transcriptional co-regulator. Hence, future work is needed to characterize the precise molecular mechanisms behind the Sl-DET1/HP2-mediated regulation of tomato fruit hormonal balance and physiology.

Although the exact mechanisms behind the light-triggered alterations in fruit hormone responsiveness are not yet clear, the data obtained in this study provide clear evidence that an intricate crosstalk between light, ethylene and auxin signaling may be involved in controlling tomato fruit carotenogenesis. Therefore, these findings open up a window of opportunity for further improvement in tomato fruit nutritional content through the combined manipulation of auxin, ethylene and light signaling-related genes.

## Author Contributions

LF, LP, MR, and AC designed the experiments. AC, RB, FA, and EP conducted the experiments and analyzed the results. AC, RB, and LF prepared the manuscript.

## Conflict of Interest Statement

The authors declare that the research was conducted in the absence of any commercial or financial relationships that could be construed as a potential conflict of interest.

## Footnotes

1 https://solgenomics.net/

2 http://plantpan2.itps.ncku.edu.tw/

3 http://jmp.com
