# Supplementary material for: Light, Ethylene and Auxin Signaling Interaction Regulates Carotenoid Biosynthesis During Tomato Fruit Ripening
Source: Front Plant Sci. 2018 Sep 18;9:1370. doi: 10.3389/fpls.2018.01370 (PMC6153336; doi:10.3389/fpls.2018.01370)
Supplement: Supplementary file 1 [file Data_Sheet_1.PDF]

Light, ethylene and auxin signaling interaction regulates carotenoid biosynthesis during tomato fruit ripening

Authors: Aline Bertinatto Cruz, Ricardo Ernesto Bianchetti, Frederico Rocha Rodrigues Alves, Eduardo Purgatto, Lazaro Eustaquio Pereira Peres, Magdalena Rossi, Luciano Freschi

The following Supplementary Information is available for this article:

Supplementary Figure 1. Overview of light influence on tomato fruit carotenogenesis.

Supplementary Figure 2. Principal component analysis (PCA) of carotenoid data.

Supplementary Figure 3. Color changes and chlorophyll levels in light- and dark-treated fruits.

Supplementary Figure 4. HY5- and PIF-binding motifs identified in the promoter regions of genes encoding master regulators of ripening.

Supplementary Figure 5. Transcript abundance of ethylene perception and signaling genes in dark- and light ripened fruits.

Supplementary Figure 6. HY5- and PIF-binding motifs identified in the promoter regions of ethylene perception and signaling genes.

Supplementary Figure 7. Transcript abundance of ripening-related tomato *Aux/IAA* genes in dark- and light ripened fruits.

Supplementary Figure 8. Transcript abundance of ripening-related tomato *ARF* genes in dark- and light ripened fruits.

Supplementary Table 1. Primer sequences used for qPCR.

Supplementary Table 2. Two-way analysis of variance (ANOVA) between genotypes, light treatment and their interactions.

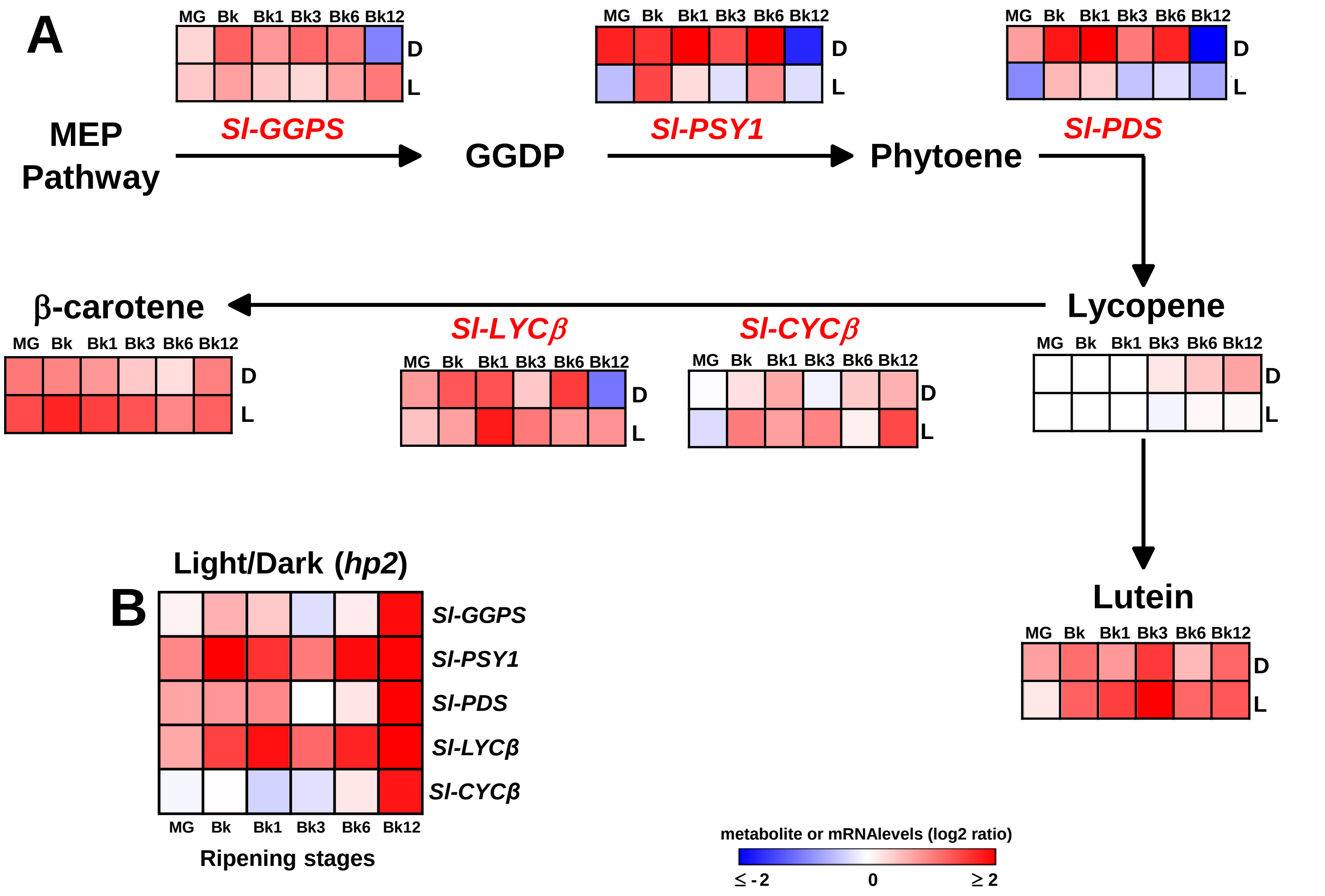

Supplementary Figure 1. Overview of light influence on tomato fruit carotenogenesis. Heat map representation of metabolites and relative mRNA levels of carotenoid biosynthesis genes. Wild-type (WT) and *high pigment-2* (*hp2*) fruits harvested at mature green (MG) stage were left to ripen under constant light (L) or dark (D) conditions. Pericarp samples were harvested at MG (two days after the beginning of treatment), breaker (Bk), Bk1 (1 day after Bk), Bk3, Bk6 and Bk12 stages. (**A**) Schematic representation of carotenoid biosynthetic pathway in tomato. Intermediate reactions are omitted. Comparison of metabolite content and relative mRNA levels of carotenoid biosynthesis genes between the WT and *hp2* fruits ripened under light or dark conditions. (**B**) Comparison of relative mRNA levels of carotenoid biosynthesis genes between light and dark samples of *hp2* fruits at each sampling time. The absolute metabolite and relative transcript values are presented in Figure 1. Data are means (±SE) of at least three biological replicates. MEP, Methylerythritol 4-phosphate; GGDP, Geranylgeranyl diphosphate; GGPS, GGDP synthase; PSY, Phytoene synthase; PDS, Phytoene desaturase; LCYβ, Chloroplast-specific β-lycopene cyclase; CYCβ, Chromoplast-specific β-lycopene cyclase.

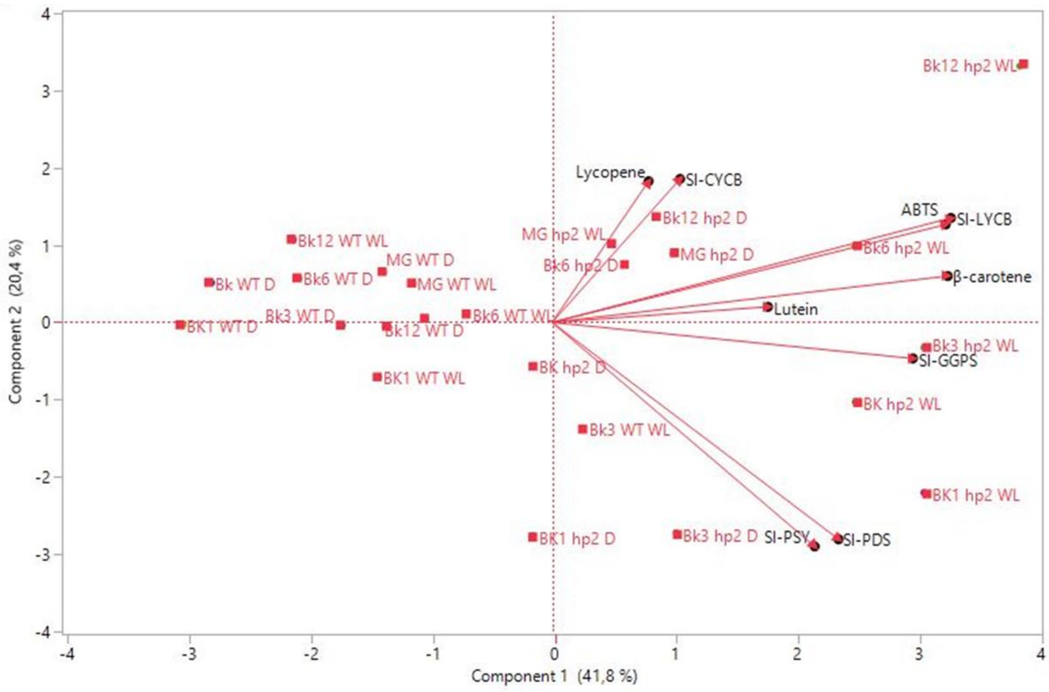

Supplementary Figure 2. Principal component analysis (PCA) of carotenoid data. The PCA plot was generated with metabolite and transcript abundance data from wild-type (WT) and *high pigment*-2 (*hp2*) fruits left to ripen under constant light (WL) or dark (D) conditions. Treatment details as described in Supplementary Figure 1. MG, mature green; Bk, breaker.

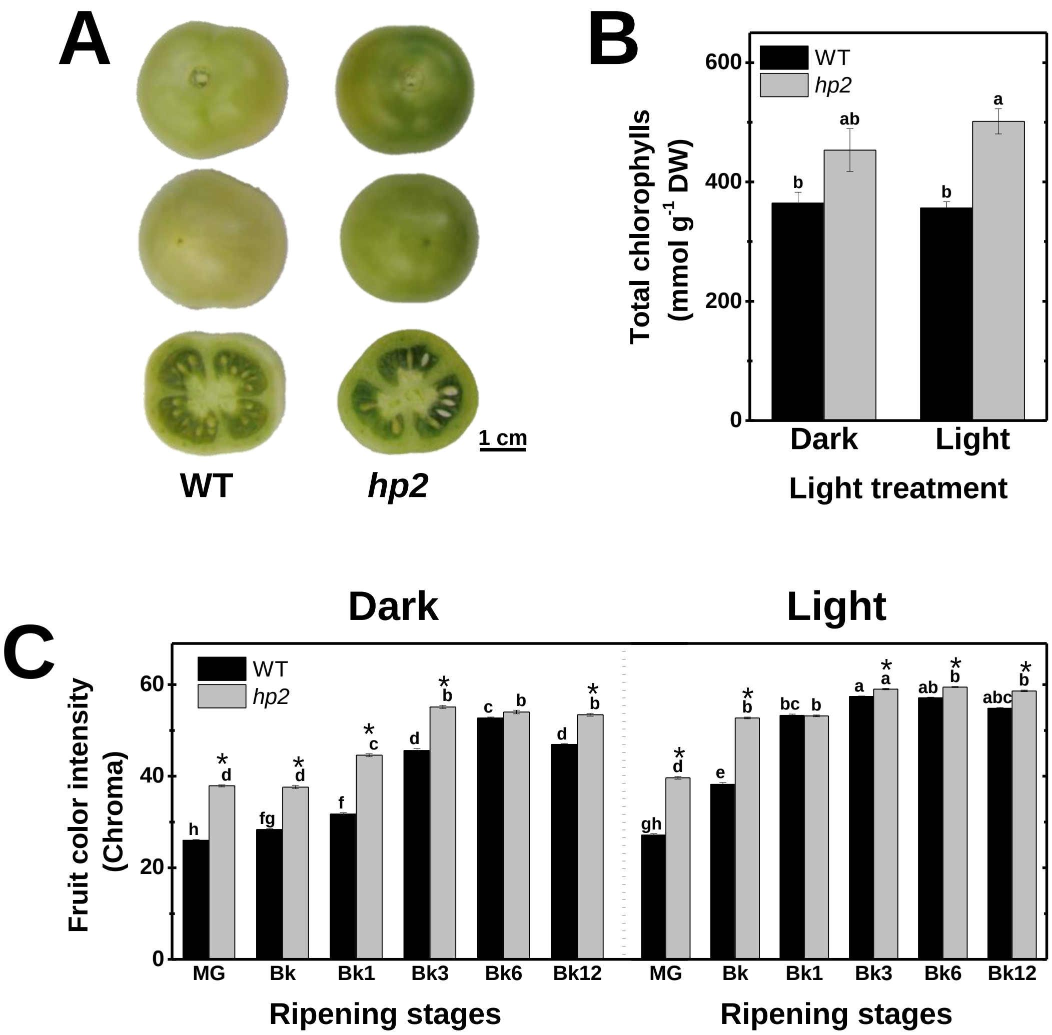

Supplementary Figure 3. Color changes and chlorophyll levels in light- and dark-treated fruits. Treatment details as described in Supplementary Figure 1. (**A**) Representative images of wild-type (WT) and *high pigment-2* (*hp2*) fruits at mature green (MG) stages. (**B**) Chlorophyll content at MG stage. (**C**) Ripening-related changes in fruit color intensity (Chroma). Data are means (±SE) of at least three biological replicates. Different letters indicate statistically significant differences (Tukey's test, $p < 0.05$) within all data (in B) or within each genotype (in C). In C, asterisks indicate statistically significant differences (Student's t-test, $p < 0.05$) between genotypes. MG, mature green; Bk, breaker.

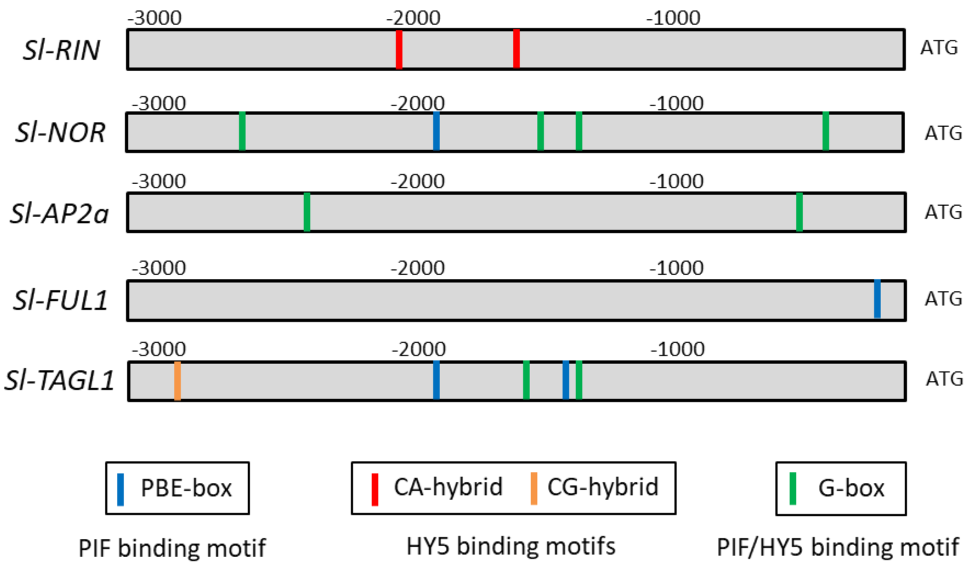

Supplementary Figure 4. HY5- and PIF-binding motifs identified in the promoter regions of genes encoding master regulators of ripening. Fragments of 3 kb upstream ATG initiation site of these genes are represented by gray bars. Motif positions are indicated by color lines: PBE-box (blue), recognized by PHYTOCHROME-INTERACTING FACTORs (PIFs). CA-hybrid (red) and CG-hybrid (yellow), recognized by ELONGATED HYPOCOTYL 5 (HY5). G-box (green), recognized by both PIFs and HY5. RIN, ripening inhibitor; NOR, non-ripening; FUL1, fruitfull1; AP2a, apetala2a; TAGL1, tomato agamous-like1.

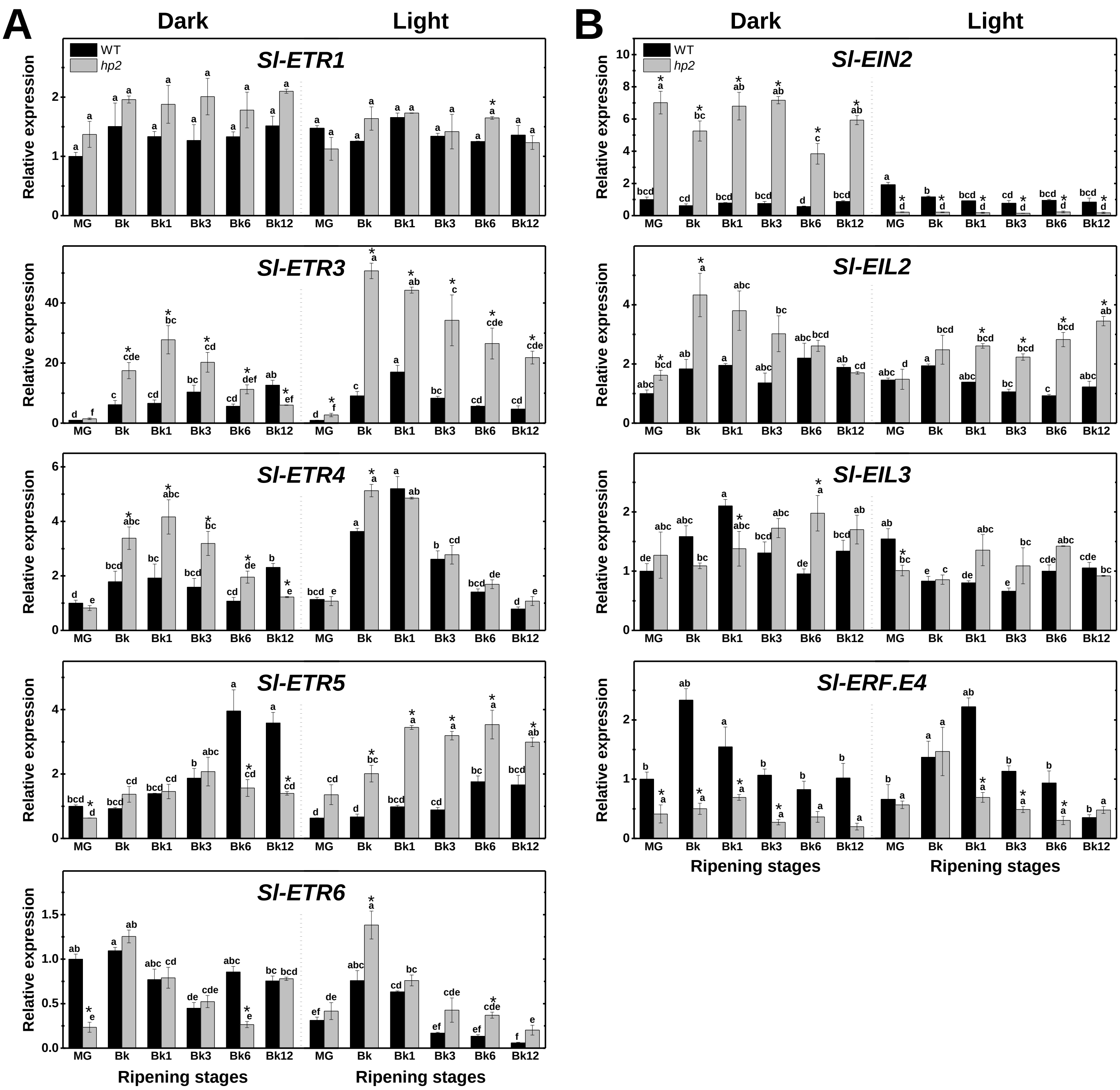

Supplementary Figure 5. Transcript abundance of ethylene perception and signaling genes in dark- and light ripened fruits. Treatment details as described in Supplementary Fig. 1. **(A)** Relative mRNA levels of tomato genes encoding ethylene receptors. **(B)** Relative mRNA levels of tomato genes encoding ripening-related ethylene signaling components. Mean relative expression was normalized against wild-type (WT) samples at mature green (MG) stage under dark conditions. Data are means (±SE) of at least three biological replicates. Different letters indicate statistically significant differences (Tukey's test, $p < 0.05$) within each genotype. Asterisks indicate statistically significant differences (Student's t-test, $p < 0.05$) between genotypes. *hp2*, *high pigment-2*; Bk, Breaker; ETR, ethylene response; EIN, ethylene insensitive; EIL, ethylene insensitive 3-like; ERF, ethylene response factor.

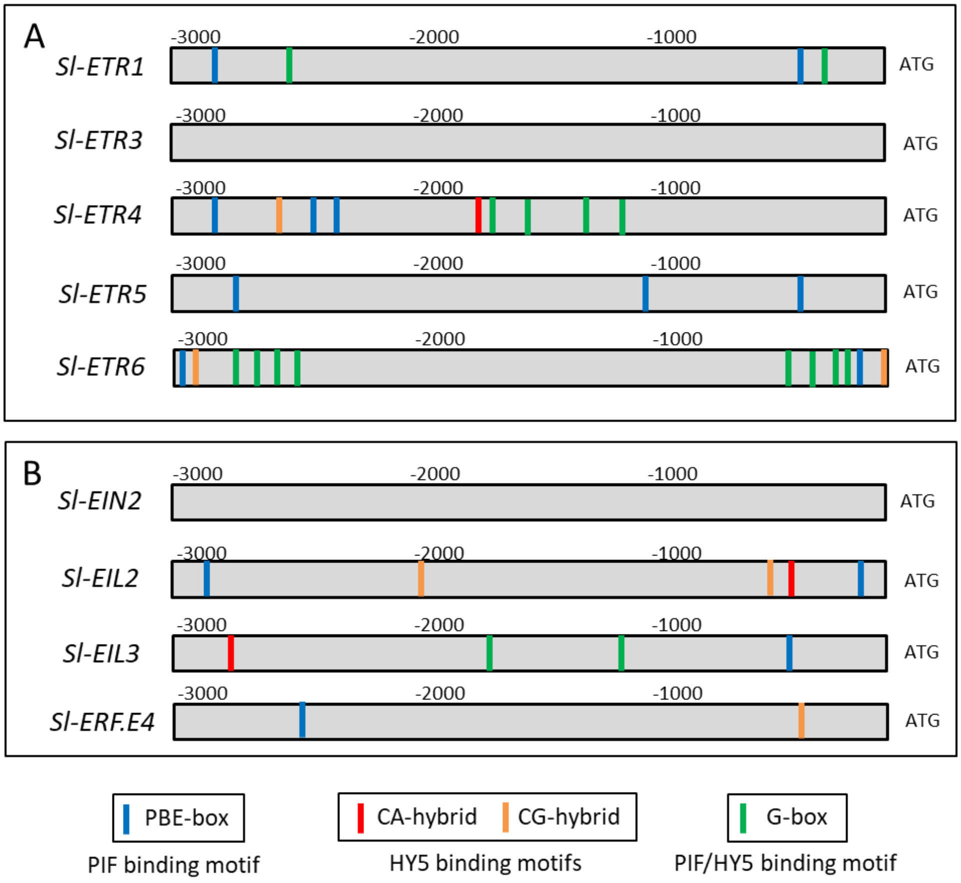

Supplementary Figure 6. HY5- and PIF-binding motifs identified in the promoter regions of ethylene perception and signaling genes. (A) Ethylene receptors. **(B)** Ethylene signal transduction components. Fragments of 3 kb upstream ATG initiation site of these genes are represented by gray bars. Motif positions are indicated by color lines: PBE-box (blue), recognized by PHYTOCHROME-INTERACTING FACTORs (PIFs). CA-hybrid (red) and CG-hybrid (yellow), recognized by ELONGATED HYPOCOTYL 5 (HY5). G-box (green), recognized by both PIFs and HY5. ETR, ethylene response; EIN, ethylene insensitive; EIL, ethylene insensitive 3-like; ERF, ethylene response factor.

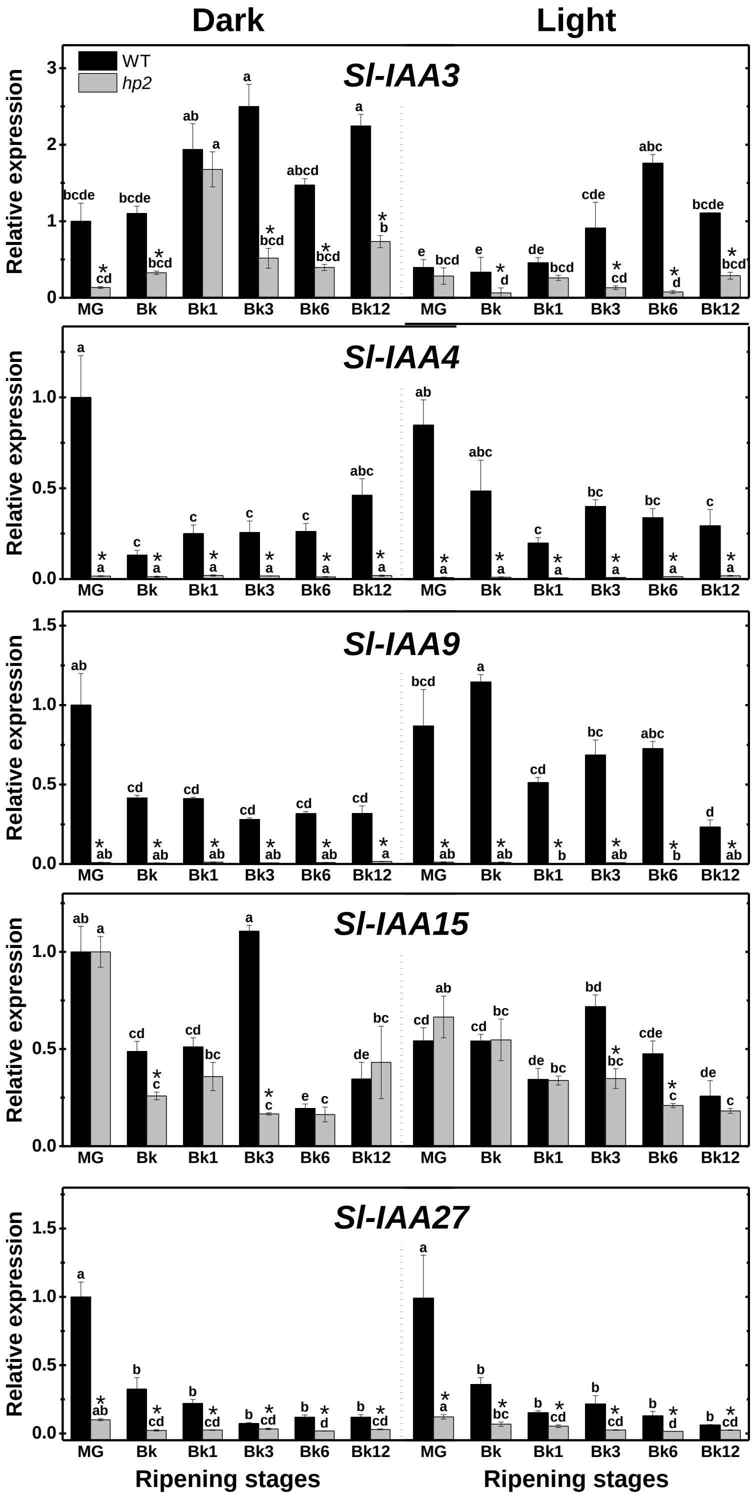

Supplementary Figure 7. Transcript abundance of ripening-related tomato *Aux/IAA* genes in dark- and light ripened fruits. Treatment details as described in Supplementary Fig. 1. Mean relative expression was normalized against wild-type (WT) samples at mature green (MG) stage under dark conditions. Data are means (±SE) of at least three biological replicates. Different letters indicate statistically significant differences (Tukey's test, $p < 0.05$) within each genotype. Asterisks indicate statistically significant differences (Student's t-test, $p < 0.05$) between genotypes. *hp2*, *high pigment-2*; Bk, Breaker; Aux/IAA, auxin/indole-3-acetic acid.

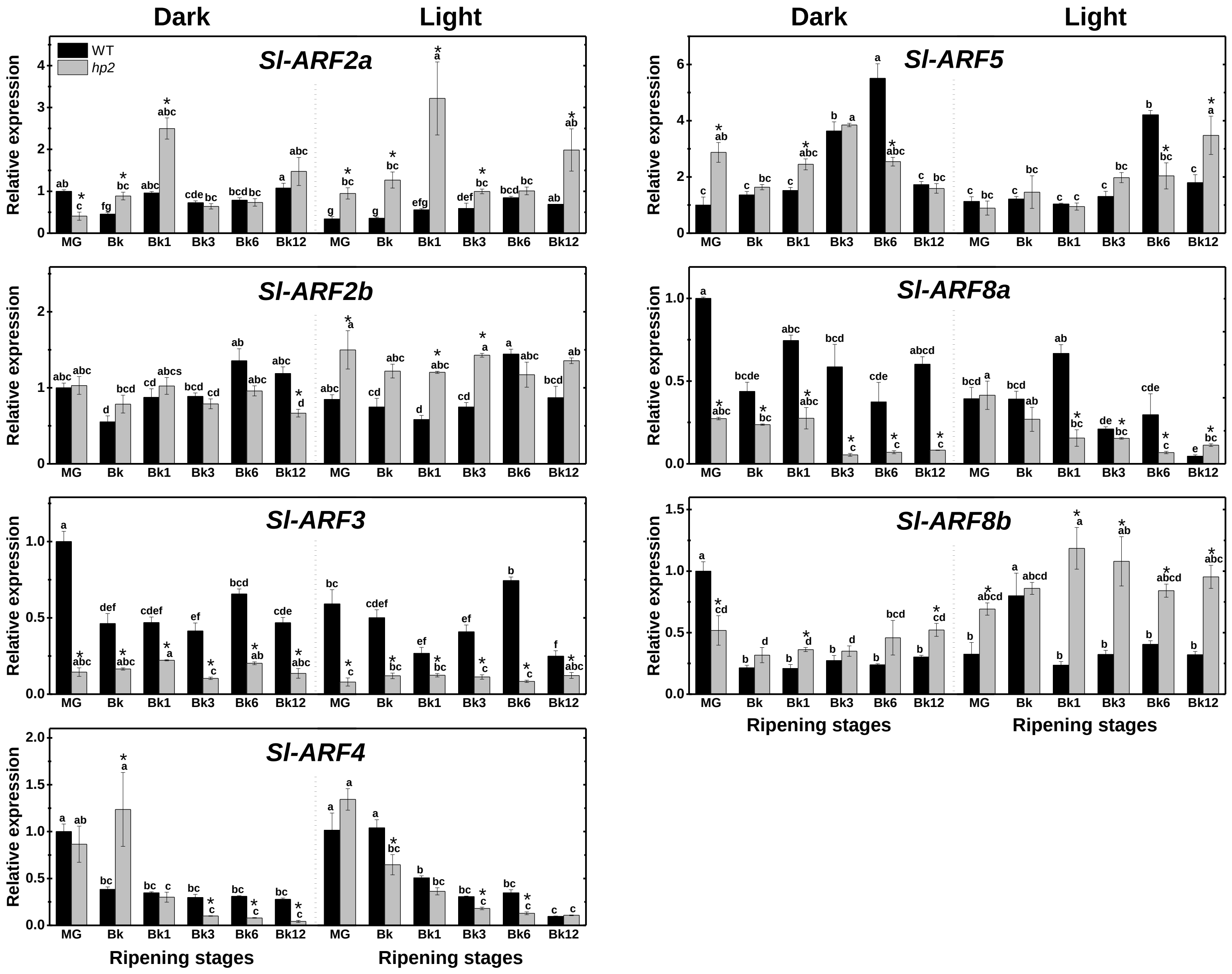

Supplementary Figure 8. Transcript abundance of ripening-related tomato *ARF* genes in dark- and light ripened fruits. Treatment details as described in Supplementary Fig. 1. Mean relative expression was normalized against wild-type (WT) samples at mature green (MG) stage under dark conditions. Data are means (±SE) of at least three biological replicates. Different letters indicate statistically significant differences (Tukey's test, $p < 0.05$) within each genotype. Asterisks indicate statistically significant differences (Student’s t-test, $p < 0.05$) between genotypes. *hp2*, *high pigment*-2; Bk, Breaker; ARF, auxin response factor.

Supplementary Table 1. Primer sequences used for qPCR.

Gene	Primers for qRT-PCR[1]	Locus[2]
Sl-RIN	F:5´- TCAAACATCATGGCATTGTGGTG- 3´/ R:5´- TGCATTTTCGGGTTGTACATTATCG- 3´	Solyc05g012020
Sl-NOR	F:5´- TAATGATGGGGTCGTCTTTCG- 3´/ R:5´- ATTTTACAGGGCTAACTATTTTTTGC- 3´	Solyc10g006880
Sl-AP2a	F:5´- AACGGACCACAATCTTGAC- 3´ / R:5´- CTGCTCGGAGTCTGAACC- 3´	Solyc03g044300
Sl-FUL1	F:5´- GTTTTGCCACAACAACTGGACTC- 3´ / R:5´- CTTGCTGCTGTGAAGAACTACC- 3´	Solyc06g069430
Sl-TAGL1	F:5´- ACTTTCTGTTCTTTGTGATGCT- 3´ / R:5´- TTGGATGCTTCTTGCTGGTAG- 3´	Solyc07g055920
Sl-GGPS	F:5´- GCTGTTGGTGTCTTATATCGTG- 3´ / R:5´- CTTCTCAATGCCATAAACGCTG- 3´	Solyc09g008920.2.1
Sl-PSY1	F:5´- CGATGGTGCTTTGTCCGATAC- 3´ / R:5´- CTCATCAACCCAACCGTACC- 3´	Solyc03g031860
Sl-PDS	F:5´- CGTTCCGTGCTTCTCCGC- 3´ / R:5´- CTAGAACATCCCTTGCCTCCAG - 3´	Solyc03g123760
Sl-LCYβ	F:5´- TTGACTTAGAACCTCGTTATTGG - 3´ / R:5´- AACAGTTCCCTTTGTCATTATCT - 3´	Solyc04g040190
Sl-CYCβ	F:5´- GCACCCACATCAAAGCCAGAG - 3´ / R:5´- GCCACATGGAGAGTGGTGAAG - 3´	Solyc06g074240
Sl-ETR1	F:5´- GGAAGAACATTGGCATTGGAAG- 3´ / R:5´- CCAACTGGATTTTGGTGTCGT- 3´	Solyc12g011330
Sl-ETR3	F:5´- TGCTGTTCGTGTACCGCTTT- 3´ / R:5´- TCATCGGGAGAACCAGAACC- 3´	Solyc09g075440
Sl-ETR4	F:5´- TGGAGGAGTGAGTGTGGATGC- 3´ / R:5´- ATGGCTGTCGTTCTTGGGC- 3´	Solyc06g053710
Sl-ETR5	F:5´- GTGCTCTGGGCCCTTCACTA- 3´ / R:5´- GAACTTACGCACCCTCAATGC- 3´	Solyc11g006180
Sl-ETR6	F:5´- TCAAAAAGCCGGTGATCTCG- 3´ / R:5´- GCACCCATTTGAACGGAAAA- 3´	Solyc09g089610
Sl-ERF.E4	F:5´- AGGCCAAGGAAGAACAAGTACAGA- 3´ / R:5´- CCAAGCCAAACGCGTACAC- 3´	Solyc01g065980
Sl-ACO1	F:5´- ACAAACAGACGGGACACGAA- 3´ / R:5´- CTCTTTGGCTTGAAACTTGA- 3´	Solyc07g049530
Sl-ACO2	F:5´- TTTATTACAAAGTGTGCGTCCCTA- 3´ / R:5´- CTCATTTTTGGGTATTAAAATATGTGT- 3´	Solyc12g005940
Sl-ACO4	F:5´- TGATCAAATTGCAAGTGCTTAAA- 3´ / R:5´- ACCACACAACAATCACACACA- 3´	Solyc02g081190
Sl-ACS1b	F:5´- TCGTTTCGAAGATTGGATGA- 3´ / R:5´- CAACAACAACAAATCTAAGCCATT- 3´	Solyc08g081550
Sl-ACS2	F:5´- GGCTACTAATGAAGAGCATGGC- 3´ / R:5´- GACCCATTTGGATAACTCCGTTG- 3´	Solyc01g095080
Sl-ACS4	F:5´- GACTCTCATACTTTGTTTGGCT- 3´ / R:5´- TTGACATTTGCTTGTTGACC- 3´	Solyc05g050010
Sl-ACS6	F:5´- CTCCTATGGTCCAAGCAAGG- 3´ / R:5´- CGACATGTCCATAATTGAACG- 3´	Solyc08g008100
Sl-EIN2	F:5´- CGATGATTGCTTTGGACCT- 3´ / R:5´- CTGCCATTGTCAAAGAGAG- 3´	Solyc09g007870
Sl-EIL2	F:5´- TGAAGATGATGGAAGTCTGTAAGG- 3´ / R:5´- CCACTCCCTGAGATTATCCGA- 3´	Solyc01g009170
Sl-EIL3	F:5´- ATCTCTCCCGACATTGCT- 3´ / R:5´- TTCCACATCGTAATCGCT- 3´	Solyc01g096810
Sl-GH3	F:5´- TATCCGCTCATCCCATCTC- 3´ / R:5´- GTATAGTCCTTTGCCTTTGTC- 3´	Solyc01g107390.2.1
Sl-ARF2a	F:5´- GCAAGGTCAAGAGTTATCGA- 3´ / R:5CATTGGTTTCTCAGACAAGTC- 3´	Solyc03g118290
Sl-ARF2b	F:5´- CTGGGTTAAGCGACAAGCTC- 3´ / R:5CCCCGCATTTGATACAGAG- 3´	Solyc12g042070
Sl-ARF3	F:5´- GATTGTTTTGCTCCCTTGGA- 3´ / R:5GTGGCTGACCCCGATAGATA- 3´	Solyc02g077560
Sl-ARF4	F:5´- TGAAAGCCATCAACTCTCGG- 3´ / R:5ATCCCATCTGACCATCAAGCATC- 3´	Solyc11g069190
Sl-ARF5	F:5´- TTCCGAGCCAAGAAAAGAAA- 3´ / R:5 -CACTCGCATCAGTTGGAAGA- 3´	Solyc04g081240
Sl-ARF8a	F:5´- AGCCCGTCCAATATGTTCAG- 3´ / R:5 -TTTGATGGTTGCTTCTGCTG- 3´	Solyc03g031970
Sl-ARF8b	F:5´- CATCTCCTTCCGACCACAGT- 3´ / R:5 -TGGTGGATCAATTTGTCCTGC- 3´	Solyc02g037530
Sl-IAA3	F:5´- GCCACCAGTTCGATCATACA- 3´ / R:5 -ATAAGGTGCTCCATCCATGC- 3´	Solyc09g065850
Sl-IAA4	F:5´- ACTCCACCTGTTGCCAAGAC- 3´ / R:5 -AGATAAGGGGCTCCATCCAT- 3´	Solyc06g053840
Sl-IAA9	F:5´- CAGAGGGGAAGTTTCTGTCG- 3´ / R:5 -CAACCTGTGCCTTTGTAGCA- 3´	Solyc04g076850
Sl-IAA15	F:5´- ATCGGAGACAGCCAAATCAG- 3´ / R:5 -TTTGCTGGAGGTTTGTTTCC- 3´	Solyc03g120390
Sl-IAA27	F:5´- GCAAGAGAAGCTCAGTGA- 3´ / R:5 -ACATCTCCCAAGGAACATCG- 3´	Solyc03g120500
Sl-CAC (constitutive)	F:5´- CCTCCGTTGTGATGTAACTGG- 3´ / R:5 -ATTGGTGGAAAGTAACATCATCG- 3´	Solyc08g006960
Sl-EXPRESSED (constitutive)	F:5´- GCTAAGAACGCTGGACCTAATG - 3´ / R:5´- TGGGTGTGCCTTTCTGAATG - 3´	Solyc07g025390

[1]F: forward, R: reverse
[2]Locus according to the Sol Genomics Network database (http://solgenomics.net/).

Supplementary Table 2. Two-way analysis of variance (ANOVA) between genotypes, light treatment and their interactions.

	Genotype *p-value*	Light treatment *p-value*	Genotype x light treatment *p-value*
Hue angle	< 0.0001*	< 0.0001*	0.0050*
Chroma	< 0.0001*	< 0.0001*	< 0.0001*
Sl-GGPS mRNA levels	< 0.0001*	< 0.0001*	0.3796
Sl-PSY mRNA levels	0.0001*	< 0.0001*	0.7294
Sl-PDS mRNA levels	< 0.0001*	< 0.0001*	< 0.0001*
Sl-LYCβ mRNA levels	< 0.0001*	< 0.0001*	< 0.0001*
Sl-CYCβ mRNA levels	< 0.0001*	0.0454*	0.0017*
Lutein content	< 0.0001*	0.1021	0.3924
β-carotene content	< 0.0001*	< 0.0001*	< 0.0001*
Lycopene content	< 0.0001*	0.0024*	< 0.0001*
Antioxidant capacity (TEAC)	< 0.0001*	0.0019*	< 0.0001*
Sl-RIN mRNA levels	< 0.0001*	0.0002*	< 0.0001*
Sl-NOR mRNA levels	< 0.0001*	0.0035*	< 0.0001*
Sl-FUL1 mRNA levels	< 0.0001*	< 0.0001*	0.0102*
Sl-AP2 mRNA levels	< 0.0001*	0.6995	0.0225*
Sl-TAGL1 mRNA levels	< 0.0001*	< 0.0001*	< 0.0001*
Endogenous ACC content	< 0.0001*	0.6373	0.0003*
ACO activity	< 0.0001*	0.0428*	0.0050*
Ethylene emission	< 0.0001*	< 0.0001*	0.0093*
EBS::GUS activity	< 0.0001*	< 0.0001*	< 0.0001*
Sl-ERF.E4 mRNA levels	< 0.0001*	0.6293	0.0050*
Sl-ACS1 mRNA levels	< 0.0001*	< 0.0001*	< 0.0001*
Sl-ACS2 mRNA levels	0.0077*	0.0399*	0.0216*
Sl-ACS4 mRNA levels	< 0.0001*	< 0.0001*	< 0.0001*
Sl-ACS6 mRNA levels	0.2637	0.3480	0.0115*
Sl-ACO1 mRNA levels	0.0022*	0.2982	0.0109*
Sl-ACO2 mRNA levels	0.3998	0.8498	< 0.0001*
Sl-ACO4 mRNA levels	0.0291*	0.0003*	< 0.0001*
Sl-ETR1 mRNA levels	0.0801	0.0549	0.0045*
Sl-ETR3 mRNA levels	< 0.0001*	< 0.0001*	< 0.0001*
Sl-ETR4 mRNA levels	< 0.0001*	< 0.0001*	0.0235*
Sl-ETR5 mRNA levels	< 0.0001*	0.1947	< 0.0001*
Sl-ETR6 mRNA levels	0.6483	< 0.0001*	< 0.0001*
Sl-EIN2 mRNA levels	< 0.0001*	< 0.0001*	< 0.0001*
Sl-EIL2 mRNA levels	< 0.0001*	< 0.0001*	0.0018*
Sl-EIL3 mRNA levels	0.1327	< 0.0001*	0.5644
Endogenous IAA content	0.0023*	< 0.0001*	0.7817
DR5::GUS activity	< 0.0001*	0.0035*	< 0.0001*
Sl-IAA3 mRNA levels	< 0.0001*	< 0.0001*	0.0021*
Sl-IAA4 mRNA levels	< 0.0001*	0.7177	0.5899
Sl-IAA9 mRNA levels	< 0.0001*	0.0001*	< 0.0001*
Sl-IAA15 mRNA levels	< 0.0001*	0.0150*	0.0316*
Sl-IAA27 mRNA levels	< 0.0001*	0.7678	0.9378

	Genotype *p*-value	Light treatment *p*-value	Genotype x light treatment *p*-value
Sl-ARF2a mRNA levels	< 0.0001*	0.1431	< 0.0001*
Sl-ARF2b mRNA levels	< 0.0001*	< 0.0001*	< 0.0001*
Sl-ARF3 mRNA levels	< 0.0001*	< 0.0001*	0.1701
Sl-ARF4 mRNA levels	0.9008	0.3884	0.0771
Sl-ARF5 mRNA levels	0.4642	< 0.0001*	0.4443
Sl-ARF8a mRNA levels	< 0.0001*	0.0877	< 0.0001*
Sl-ARF8b mRNA levels	< 0.0001*	< 0.0001*	< 0.0001*

Asterisks indicate statistically significant differences ($P < 0.05$).
